# Socioeconomic background and childhood cancer survival in Germany: A nationwide assessment based on data from the German Childhood Cancer Registry

**DOI:** 10.1002/ijc.70042

**Published:** 2025-07-17

**Authors:** Maike Wellbrock, Arndt Borkhardt, Cécile M. Ronckers, Claudia Spix, Desiree Grabow, Anna‐Liesa Filbert, Daniel Wollschläger, Friederike Erdmann

**Affiliations:** ^1^ Research Group Aetiology and Inequalities in Childhood Cancer, Division of Childhood Cancer Epidemiology Institute of Medical Biostatistics, Epidemiology and Informatics (IMBEI), University Medical Center of the Johannes Gutenberg University Mainz Germany; ^2^ Department of Prevention and Evaluation Leibniz Institute for Prevention Research and Epidemiology—BIPS Bremen Germany; ^3^ Department of Paediatric Oncology, Haematology and Clinical Immunology Heinrich Heine University, Medical Faculty Düsseldorf Germany; ^4^ German Cancer Consortium (DKTK), Partnering Site Essen/Düsseldorf Düsseldorf Germany; ^5^ Division of Childhood Cancer Epidemiology/German Childhood Cancer Registry Institute of Medical Biostatistics, Epidemiology and Informatics (IMBEI), University Medical Center of the Johannes Gutenberg University Mainz Germany; ^6^ Division of Epidemiological Methodology and Radiation Research Institute of Medical Biostatistics, Epidemiology and Informatics (IMBEI), University Medical Center of the Johannes Gutenberg University Mainz Germany

**Keywords:** childhood cancer survival, Germany Childhood Cancer Registry, social inequalities, socioeconomic deprivation

## Abstract

Social inequalities in childhood cancer survival have been observed in many countries, including European nations with universal healthcare systems, suggesting that not all children with cancer have benefited equally from diagnostic and therapeutic enhancements. Despite the growing socioeconomic diversity within Germany's large population, little is known about the extent of social inequalities in German childhood cancer survival. Using German Childhood Cancer Registry data, we identified all children with a cancer diagnosis before the age of 15 years in 1997–2016 in Germany (*N* = 35,443). Based on individual residential address information (at time of diagnosis) we applied the German Index of Socioeconomic Deprivation (GISD) to measure area‐based socioeconomic status. Using Cox proportional hazards models, we assessed the association between absolute area‐based socioeconomic deprivation (AASD) and 10‐year overall survival (OS) (end of follow‐up: 15 January 2023) to estimate hazard ratios (HR) and corresponding 95% confidence intervals (CI). The multivariable analyses revealed a null association for AASD and 10‐year OS for all cancers combined (HR_adj_ = 1.00, 95% CI 0.97; 1.03). Among children diagnosed with acute myeloid leukaemia and germ cell tumors, a higher AASD (higher levels of deprivation) appeared to be associated with worse survival, particularly pronounced in boys. The opposite was observed among children diagnosed with central nervous system tumors. Contrary to reports from other European countries, we found little evidence for social inequalities in childhood cancer survival in Germany when analysing the GISD. Further research assessing individual‐level measures of socioeconomic status is warranted.

## What's New?

Significant advances in childhood cancer survival have transformed outcomes for many children globally. However, emerging evidence suggests that, even in settings with universal healthcare, these gains are not equally distributed. Here, the authors focused on childhood cancer survival in Germany, using data from the German Childhood Cancer Registry to identify associations with area‐based socioeconomic status (SES) measures that encompass defined geographical regions. Overall, the idea that social inequalities are associated with childhood cancer survival in Germany was largely unsupported. Future investigations are needed to determine whether other SES dimensions, particularly family SES, impact survival patterns among children with cancer.

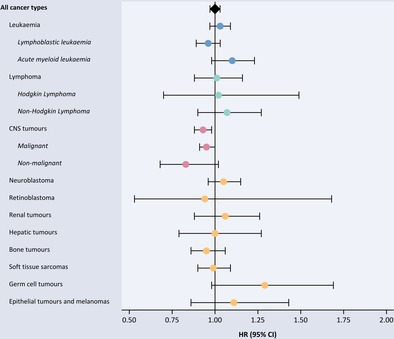

**Abbreviations**
AASD: absolute area‐based socioeconomic deprivation
ALL: acute lymphoblastic leukaemia
AML: acute myeloid leukaemia
CI: confidence interval
DAG: directed acyclic graph
GCCR: German Childhood Cancer Registry
GDR: German Democratic Republic
GISD: German Index of Socioeconomic Deprivation
HIC: high‐income country
HL: Hodgkin lymphoma
HR: hazard ratio
ICCC‐3: International Classification of Childhood Cancer—third edition
ICD‐O‐3: International Classification of diseases for Oncology—third edition
LL: lymphoblastic leukaemia
N: number
NHL: non‐Hodgkin lymphoma
OS: overall survival
RKI: Robert Koch Institute
SES: socioeconomic status
SPN: subsequent primary neoplasm
UK: United Kingdom
US: United States

## 1 BACKGROUND

Advances in diagnostics and treatment of childhood cancer, including the use of highly standardised treatment protocols, risk‐adapted therapy allocation, and a greater focus on supportive care, have led to substantial enhancements in survival.^1^, ^2^, ^3^ During the last six decades, five‐year survival has improved substantially, from approximately 30% in the 1960s to exceeding 85% in some high‐income countries (HICs) today.^1^, ^3^, ^4^

However, not all children seem to have benefitted equally from these improvements.^5^, ^6^ Beyond well‐established clinical factors, evidence is accumulating that socioeconomic conditions may be associated with prognosis, even in European countries where equal access to healthcare services is presumed.^5^, ^7^, ^8^, ^9^ Hence, social survival inequalities exist not only between countries with different levels of socioeconomic development, but also within HICs.^6^ Overall, research investigating such survival inequalities indicated disadvantages for children of families with lower socioeconomic status (SES). However, observations vary, at least to some extent, across countries, by tumour type and by the particular SES measures employed.^5^, ^10^, ^11^, ^12^, ^13^, ^14^

Based on a population register infrastructure with longstanding high‐quality data on socioeconomic circumstances and health, the association between SES and childhood cancer survival has been investigated particularly well in the Nordic countries, where it has indicated disadvantages for children of families with low incomes, poor living conditions, and shorter parental education, and those with only one caregiver in the household.^5^, ^8^, ^10^, ^11^, ^15^ Notably, these countries are widely regarded as among the most egalitarian in the world, with extensive welfare systems, universal tax‐financed healthcare services, and a tradition of social justice policies. Thus, social inequalities in Nordic countries are usually not as pronounced as in other parts of Europe, in particular the south‐eastern European region.^16^, ^17^ This leaves the concern that larger social inequalities may come with larger survival inequalities in countries with a wider socioeconomic heterogeneity, such as Germany. Those larger social inequalities may result from the continuing combined effects of the socioeconomic East–West gradient of the German Reunification in 1990, a larger number of migrants in the population, and higher (child) poverty, among other factors.^18^, ^19^

While multiple studies revealed SES‐related survival inequalities for various cancer types among adults in Germany,^20^, ^21^, ^22^ knowledge about similar survival inequalities among children is largely lacking, with the exception of children diagnosed with acute lymphoblastic leukaemia (ALL) in the early 1990s.^23^, ^24^ Evidence for social inequalities in cancer survival among German adults and among children from other European countries raises the question of whether similar survival inequalities affect children in Germany. A better understanding may shed light on mechanisms underlying observed survival patterns. However, assessing social survival inequalities is challenging; cancer in children is comparatively rare and encompasses a heterogeneous group of specific, even less frequent cancer types.^4^, ^25^, ^26^, ^27^ Furthermore, robust information on SES is required.

In the present study, we leveraged the high‐quality data of the German Childhood Cancer Registry (GCCR)^25^ and the possibility of assigning area‐based socioeconomic background scores to assess the presence and patterns of social inequalities in childhood cancer survival in Germany.

## 2 METHODS

### 2.1 Study population

We identified all children recorded in the GCCR who were diagnosed with cancer before the age of 15 years between 1997 and 2016, while residing in Germany at the time of diagnosis. The GCCR monitors incident diagnoses of cancers in children with nationwide coverage and a high degree of completeness, estimated to exceed 95%.^25^ On average, approximately 1850 incident cases are reported each year from a population of around 11 million children below the age of 15 years in Germany. We considered only the first cancer diagnosis for each child and neglected any subsequent primary neoplasms (SPN) occurring in the eligible age group (0–14 years) in the analysis. Diagnoses were categorised according to the nomenclature of the International Classification of Childhood Cancer, third edition (ICCC‐3),^28^ which classifies tumors according to the International Classification of Diseases for Oncology, third edition (ICD‐O‐3), into 12 major diagnostic groups and 47 subgroups. In addition to the diagnostic information, basic demographic data are recorded in the GCCR, including place of residence at diagnosis.

The GCCR collects information on vital status at least every 2 years using information from the relevant therapy trials, paediatric haematology‐oncology units, and local population registries. For the present study, we used the GCCR database as of 15 January 2023.

We excluded patients with no follow‐up information (*N* = 415; 1.2%) or missing residential address information (*N* = 108; 0.3%), resulting in an analytical sample of 35,443 children (Figure S1).

### 2.2 Operationalisation of socioeconomic measures

Individual SES is a multidimensional construct,^29^ describing both the material and social resources and assets as well as the individual's rank or position within a social hierarchy.^29^ SES cannot be measured directly, but by its contributing indicators at different structural levels: at the individual level (such as by education or occupation), at the household level (such as by the household/family income or savings) or at the area or neighbourhood level (such as by area deprivation index or available facilities).^29^, ^30^ Since individual SES measures are challenging to assess retrospectively in epidemiological research, an area‐based measure is commonly used. Area‐based SES metrics take into account various socioeconomic indicators estimated for the residents of a given region and do not refer to an individuals' SES.^31^

For the present study, we used the German Index of Socioeconomic Deprivation (GISD) as an indicator of the area‐based socioeconomic background (exposure), which can be seen as one of the SES dimensions mentioned above. The GISD was developed and published by the Robert Koch Institute (RKI) in Berlin, Germany, the government's central scientific institution in the field of biomedicine, and is intended to cover the overall set of socioeconomic advantages and disadvantages of a defined geographical area in Germany on different spatial levels.^32^ The latest version of the GISD (as of December 2022) is based on nine indicators describing the social conditions of individuals and households in a given municipality. These nine indicators correspond to three dimensions of SES: income, occupation and education. The annual GISD scores are available from 1998 to 2019, with biennial updates. They are normalised to values between 0 and 1 per calendar year, reflecting the ranking of a region relative to all other regions. Thus, for a given year, the least deprived region in the nation has a score of 0, and the most deprived region has a score of 1, irrespective of the absolute level of deprivation.^32^ This characteristic renders comparisons of GISD scores between years difficult and challenges the evaluation of time trends. To enable longitudinal comparisons, we recalculated the GISD without the normalisation step (Figure S7). The resulting score with values between 0 and 3 is hereinafter called Absolute Area‐based Socioeconomic Deprivation (AASD), with higher values indicating more severe levels of deprivation, representing lower area‐based SES.

We defined municipality‐level AASD as the main exposure of interest. We assigned AASD values to each cancer patient based on the eight‐digit municipality code (Official Municipality Key, AGS) of the place of residence at time of diagnosis. For the diagnostic year 1997, we applied the corresponding values of 1998. Further, we considered three single SES indicators: net household income, unemployment rate and proportion of employees with a university degree. These variables were singled out as, within the principal component analysis conducted by the RKI, they had the highest weights among all indicators for the respective underlying GISD dimensions—income, occupation and education^32^—and are also commonly used SES indicators in epidemiological research. ‘Household net income’ was defined as average monthly household income per inhabitant in EUR, and ‘Unemployment rate’ was defined as the number of unemployed individuals per 1000 inhabitants of working age. ‘Employees with a university degree’ was defined as the proportion of employed individuals subject to social insurance contributions at place of residence with a university degree as share (%) of total employees subject to social insurance contributions at place of residence.^32^

### 2.3 Statistical analysis

We defined 10‐year overall survival (OS) as our main outcome of interest. Patients were followed from the date of their cancer diagnosis until death from any cause, emigration, end of the 10‐year follow‐up, or 15 January 2023, whichever came first. Whereas 5‐year follow‐up was completed by 95.8% of the included children, 10‐year follow‐up was completed by 72.0%. Censoring at the end of follow‐up considers the condition that some patients could not reach complete follow‐up as the period between end of study (end of 2016) and date of data extraction was almost 7 years.

For descriptive presentation, absolute numbers and proportions as well as person‐years of follow‐up of all eligible incident children by cancer type, age at diagnosis, sex, diagnostic period, birth cohort, and place of residence at time of diagnosis (urban/rural) were reported. In each of these subgroups, we compared the arithmetic mean of the AASD scores of children who died within 10 years after diagnosis to those of children who were alive at the end of follow‐up. We calculated 10‐year OS using the Kaplan Meier method. To summarise the association between AASD and 10‐year OS graphically, locally estimated scatterplot smoothing (LOESS) was applied to the survival estimates displayed by the 25 percentiles of AASD.

We fitted univariable and multivariable (defined as main analyses) Cox proportional hazard models to examine the association between the different socioeconomic measures at the year of diagnosis (AASD, GISD, household net income, unemployment rate and employees with a university degree) and childhood cancer survival. Time since diagnosis was applied as the underlying time scale. Covariates included in the multivariable models were determined a priori based on a directed acyclic graph (DAG, see Figure S2), reflecting conceptual considerations about potential confounding and mediating factors. The following covariates were identified and included in the adjusted analyses: year of diagnosis, year of birth and place of residence (urban/rural). Results were expressed as Hazard Ratios (HR) with two‐sided 95% confidence intervals (CI). To check the proportional hazards assumption of the Cox regression, we carried out tests based on scaled Schoenfeld residuals. When the results were statistically significant, we visually assessed whether the trend of complementary log–log survival with log‐time was linear and parallel in groups defined by the covariate.

As an additional analysis to investigate potential geographical differences, we fitted Cox models restricted to the Western German federal states.

We used fractional polynomial regression (α = 0.05) for all numerical SES measures to detect possibly non‐linear associations with survival.^33^ The fractional polynomial regression revealed linear slopes for all tested models, except for the proportion of employees with a university degree, for which X^(−1)^ was the best‐fitting model. We used the transformed variable X^(−1)^ for analysis accordingly.

Variables included in the German Index of Deprivation had different levels of spatial resolution, leading to possibly clustered data. Therefore, the multivariable Cox models were fit with robust standard errors by using a sandwich estimator.^34^

HRs for AASD are reported per 0.3 units, based on the standard deviation (0.29) and interquartile range (0.38) of the study population's AASD. HRs for the GISD are reported accordingly per 0.2 units. HRs for net household income are reported per EUR 250, HRs for the unemployment rate per 40 (persons per 1000), and HRs for the X^(−1)^‐transformed variable of employees with a university degree per 0.05%^(−1)^. The distributions of all applied SES measures are given in Figure S3A–E.

Statistical analyses were performed using SAS Statistical Software 9.4^35^ and STATA 15.^36^

## 3 RESULTS

Among the 35,443 childhood cancer patients included in the present study, slightly over half were male (55.4%) and most of the children were diagnosed at ages 1–4 years (Table 1). The most frequent diagnoses included leukaemias (33.3%), CNS tumours (23.9%) and lymphomas (11.1%) (Table 1).

**TABLE 1:** Baseline characteristics, area‐based socioeconomic deprivation (AASD),^a^ and 10‐year overall survival of the study population of children diagnosed with a first cancer at ages 0–14 years between 1997 and 2016 in Germany.

	All children			Children alive at end of follow‐up^b^			Children deceased during follow‐up^b^			10‐year OS (95% CI)^b^	Person‐years of follow‐up^b^
	*N*	%^c^	Mean AASD^d^ (min; max)	*N*	%^e^	Mean AASD^d^ (min; max)	*N*	(%)^e^	Mean AASD^d^ (min; max)		
Diagnostic group/cancer type^f^											
All cancer types	35,443	100.0	1.50 (0.39; 2.44)	29,290	82.6	1.49 (0.39; 2.44)	6153	17.4	1.52 (0.43; 2.44)	82.1% (81.7; 82.5)	273,306.3
Leukaemia	11,794	33.3	1.49 (0.43; 2.44)	10,208	86.6	1.49 (0.43; 2.44)	1586	13.5	1.54 (0.48; 2.33)	86.3% (85.6; 86.9)	95,950.0
Lymphoblastic leukaemia	9293	26.2	1.49 (0.43; 2.44)	8325	89.6	1.49 (0.43; 2.44)	968	10.4	1.52 (0.56; 2.33)	89.3% (88.6; 89.9)	78,230.9
Acute myeloid leukaemia	1510	4.3	1.51 (0.49; 2.30)	1085	71.9	1.48 (0.50; 2.26)	425	28.2	1.57 (0.49; 2.30)	71.4% (69.0; 73.6)	10,408.9
Lymphoma	3948	11.1	1.51 (0.44; 2.31)	3665	92.8	1.50 (0.44; 2.31)	283	7.2	1.55 (0.75; 2.31)	92.6% (91.7; 93.4)	32,827.5
Hodgkin Lymphoma	1695	4.8	1.51 (0.44; 2.31)	1657	97.8	1.50 (0.44; 2.31)	38	2.2	1.58 (0.94; 2.31)	97.6% (96.7; 98.2)	14,470.3
Non‐Hodgkin Lymphoma	1437	4.1	1.50 (0.45; 2.29)	1256	87.4	1.49 (0.45; 2.29)	181	12.6	1.56 (0.75; 2.18)	87.1% (85.2; 88.8)	11,498.7
CNS tumours	8485	23.9	1.50 (0.43; 2.40)	6343	74.8	1.49 (0.46; 2.40)	2142	25.2	1.50 (0.43; 2.32)	73.7% (72.7; 74.6)	59,843.3
Malignant	4982	14.1	1.49 (0.43; 2.40)	2974	59.7	1.49 (0.46; 2.40)	2008	40.3	1.50 (0.43; 2.32)	58.3% (56.9; 59.7)	30,001.9
Non‐malignant	3503	9.9	1.50 (0.49; 2.38)	3369	96.2	1.50 (0.49; 2.37)	134	3.8	1.52 (0.82; 2.09)	95.8% (95.0; 96.4)	29,841.4
Neuroblastoma	2563	7.2	1.49 (0.45; 2.30)	1949	76.0	1.49 (0.45; 2.30)	614	24.0	1.51 (0.59; 2.26)	75.3% (73.5; 77.0)	18,714.5
Retinoblastoma	782	2.2	1.49 (0.49; 2.24)	765	97.8	1.49 (0.49; 2.24)	17	2.2	1.52 (1.23; 1.99)	97.7% (96.4; 98.6)	6731.8
Renal tumours	1950	5.5	1.51 (0.43; 2.37)	1792	91.9	1.51 (0.43; 2.37)	158	8.1	1.54 (0.54; 2.29)	91.7% (90.4; 92.9)	16,571.3
Hepatic tumours	441	1.2	1.45 (0.44; 2.32)	333	75.5	1.43 (0.44; 2.32)	108	24.5	1.51 (0.59; 2.08)	74.0% (69.4; 78.0)	2854.4
Bone tumours	1542	4.4	1.50 (0.46; 2.39)	1071	69.5	1.50 (0.46; 2.39)	471	30.5	1.50 (0.49; 2.33)	68.5% (66.1; 70.8)	10,751.5
Soft tissue sarcomas	2100	5.9	1.50 (0.39; 2.44)	1488	70.9	1.50 (0.39; 2.37)	612	29.1	1.51 (0.62; 2.44)	69.8% (67.7; 71.8)	14,396.6
Germ cell tumours	1153	3.3	1.48 (0.47; 2.29)	1083	93.9	1.48 (0.47; 2.29)	70	6.1	1.53 (0.75; 2.21)	93.7% (92.1; 95.0)	9746.7
Epithelial tumours and melanomas	635	1.8	1.47 (0.45; 2.28)	557	87.7	1.46 (0.45; 2.28)	78	12.3	1.55 (0.68; 2.13)	86.5% (83.4; 89.1)	4607.6
Other unspecified neoplasms	50	0.1	1.46 (0.94; 1.98)	36	72.0	1.44 (0.94; 1.91)	14	28.0	1.49 (0.95; 1.98)	68.7% (52.4; 80.5)	311.2
Age at diagnosis											
<1 year	3666	10.3	1.48 (0.43; 2.37)	3006	82.0	1.47 (0.43; 2.37)	660	18.0	1.52 (0.52; 2.27)	81.5% (80.2; 82.7)	27,325.9
1–4 years	12,325	34.8	1.49 (0.43; 2.33)	10,384	84.3	1.49 (0.43; 2.33)	1941	15.8	1.51 (0.43; 2.30)	83.8% (83.1; 84.4)	97,045.5
5–9 years	9373	26.5	1.50 (0.39; 2.44)	7761	82.8	1.50 (0.39; 2.44)	1612	17.2	1.51 (0.49; 2.26)	82.3% (81.5; 83.1)	73,761.4
10–14 years	10,079	28.4	1.50 (0.44; 2.44)	8139	80.8	1.49 (0.44; 2.39)	1940	19.3	1.53 (0.48; 2.44)	80.0% (79.2; 80.8)	75,173.6
Sex											
Female	15,827	44.7	1.50 (0.43; 2.44)	13,188	83.3	1.49 (0.43; 2.40)	2639	16.7	1.51 (0.48; 2.44)	82.8% (82.2; 83.4)	122,512.2
Male	19,616	55.4	1.50 (0.39; 2.44)	16,102	82.1	1.49 (0.39; 2.44)	3514	17.9	1.53 (0.43; 2.33)	81.5% (80.9; 82.0)	150,794.2
Diagnostic period											
1997–2001	9188	25.9	1.65 (0.86; 2.39)	7269	79.4	1.65 (0.86; 2.39)	1890	20.6	1.66 (0.94; 2.29)	79.1% (78.2; 79.9)	74,529.5
2002–2006	8918	25.2	1.60 (0.75; 2.44)	7263	81.4	1.60 (0.75; 2.44)	1655	18.6	1.60 (0.75; 2.44)	81.2% (80.4; 82.0)	74,659.0
2007–2011	8716	24.6	1.45 (0.53; 2.28)	7315	83.9	1.45 (0.53; 2.28)	1401	16.1	1.46 (0.59; 2.13)	83.7% (82.9; 84.4)	72,460.4
2012–2016	8621	24.3	1.27 (0.39; 2.06)	7421	86.1	1.27 (0.39; 2.06)	1200	13.9	1.26 (0.43; 1.95)	84.3% (83.3; 85.3)	51,657.3
Birth cohort											
1982–1989	3251	9.2	1.69 (0.83; 2.39)	2503	77.0	1.69 (0.83; 2.39)	748	23.1	1.69 (0.94; 2.31)	76.3% (74.8; 77.7)	24,876.9
1990–1999	14,177	40.0	1.58 (0.57; 2.44)	11,486	81.0	1.58 (0.57; 2.44)	2691	19.0	1.58 (0.62; 2.44)	80.8% (80.1; 81.4)	117,068.9
2000–2009	14,130	39.9	1.43 (0.39; 2.37)	11,962	84.7	1.43 (0.39; 2.37)	2168	15.3	1.44 (0.48; 2.30)	84.2% (83.5; 84.8)	109,044.2
2010–2016	3885	11.0	1.27 (0.43; 1.99)	3339	86.0	1.27 (0.43; 1.99)	546	14.1	1.27 (0.43; 1.95)	84.4% (83.0; 85.7)	22,316.3
Place of residence at diagnosis											
Urban	24,356	68.7	1.51 (0.43; 2.40)	20,092	82.5	1.51 (0.43; 2.40)	4264	17.5	1.53 (0.43; 2.33)	81.9% (81.4; 82.4)	186,886.0
Rural	11,087	31.3	1.46 (0.39; 2.44)	9198	83.0	1.45 (0.39; 2.44)	1889	17.0	1.49 (0.48; 2.44)	82.4% (81.7; 83.1)	86,420.4

Abbreviations: AASD, absolute area‐based socioeconomic deprivation; CI, confidence interval; CNS, central nervous system; max, maximum; min, minimum; *N*, number; OS, overall survival.

^a^ Higher AASD values indicate higher deprivation.

^b^ Children were followed from the date of cancer diagnosis until their death from any cause, emigration, the end of the 10‐year follow‐up or 15 January 2023, whichever came first.

^c^ Refers to the proportions of the column.

^d^ Refers to the AASD at the residential address at the time of diagnosis.

^e^ Refers to the proportions of the row.

^f^ Cancer diagnoses were classified according to the International Classification of Childhood Cancer—third edition (ICCC‐3).

### 3.1 Area‐based socioeconomic deprivation

GISD scores and AASD scores were strongly correlated (Spearman's rank correlation coefficient = 0.7). The values for each dimension (income, occupation and education) contributing to the annual AASD scores are given in Figure S4.

The arithmetic mean of AASD values was 1.50, ranging between 0.39 and 2.44 (Table 1, Figure S3A). The mean value of the AASD declined over time in both our study population and in all of Germany (Table 1, Figure S5). Children who had died during follow‐up had an AASD mean of 1.52, while the AASD mean for children alive at the end of follow‐up was 1.49 (Table 1). The highest AASD means were observed for deceased children diagnosed with Hodgkin lymphoma (HL) and non‐Hodgkin lymphoma (NHL) (1.58 and 1.56, respectively). The most pronounced difference between deceased and surviving children was seen in AML, HL and epithelial tumours and melanomas, with a difference in AASD of ≥0.8 (Table 1).

Checking for violation of the proportional hazards assumption resulted in 9/203 (4%) statistically significant tests. Visual inspection of complementary log–log plots showed linear and parallel trends for nearly all of those nine subgroups.

### 3.2 Association between absolute area‐based socioeconomic deprivation and survival

**FIGURE 1:**
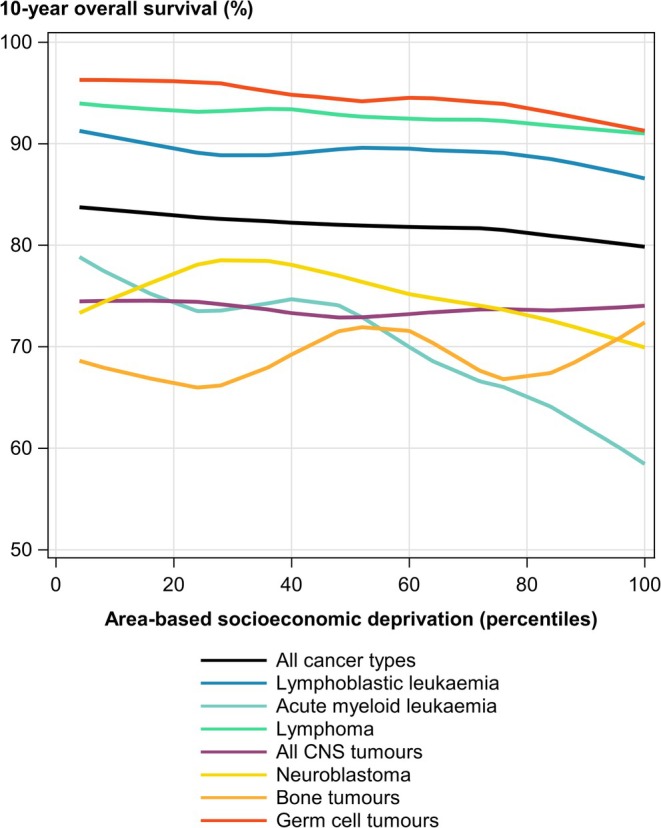
Ten‐year overall survival from childhood cancer in relation to area‐based socioeconomic deprivation (AASD) in Germany (1997–2016) by cancer type. To summarise the association between 10‐year OS and AASD graphically, a locally estimated scatterplot smoothing (LOESS) was applied to the survival estimates by 25 percentiles of AASD.

For the entire diagnostic period 1997–2016, 10‐year OS from all cancers combined was 82.1%, based on 35,443 cases and 6153 deaths. Survival varied substantially between diagnostic groups and cancer types, with the highest survival seen for children with retinoblastoma and the lowest for patients with malignant CNS tumours. Overall, survival probabilities improved over time (Table 1). Analysing AASD and OS in the univariable setting indicated decreasing survival with increasing AASD for all cancers combined (Figure 1). This was also confirmed by the crude HR of 1.07 (95% CI 1.05; 1.10) from the Cox analyses (Table S1). We observed the most pronounced associations of higher AASD and lower OS among children diagnosed with AML (HR = 1.27, 95% CI 1.15; 1.40), HL (HR = 1.26, 95% CI 0.91; 1.75), NHL (HR = 1.22, 95% CI 1.05; 1.41), hepatic tumours (HR = 1.23, 95% CI 1.01; 1.50) and epithelial tumours and melanomas (HR = 1.31, 95% CI 1.05; 1.63). The crude HRs by diagnostic period (for all cancer types combined) did not give indications of a temporal trend (Table S1).

**FIGURE 2:**
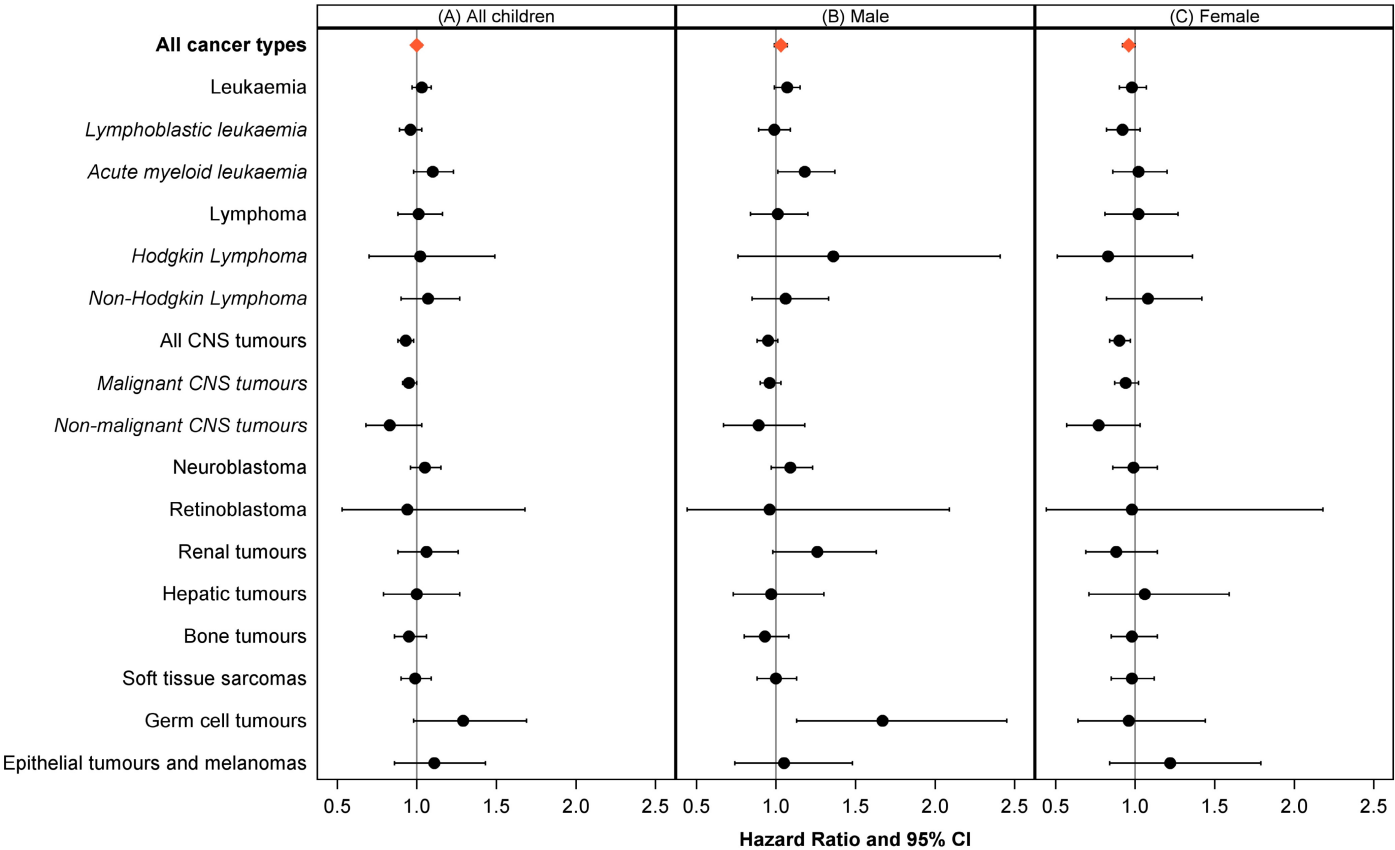
Adjusted hazard ratios with 95% confidence intervals (CI) for the association between area‐based socioeconomic deprivation (AASD) and 10‐year overall survival from childhood cancer in Germany (1997–2016) for (A) all children, (B) males and (C) females. Hazard ratios are expressed per 0.3 units AASD in accordance with the standard deviation/interquartile range. Hazard ratios were adjusted for year of diagnosis, year of birth, and place of residence (urban/rural). Abbreviations: CI, confidence interval; CNS, central nervous system.

In contrast to the crude HRs, the multivariable Cox analyses (adjusted for year of diagnosis, year of birth and place of residence) revealed a null association between AASD and OS from all cancers combined (HR_adj_ = 1.00, 95% CI 0.97; 1.03) (Table 2, Figure 2A). For a small number of cancer types, we observed indications of worse survival for children who resided in regions with higher AASD: HR_adj_ = 1.10 (95% CI 0.98; 1.23) for AML and HR_adj_ = 1.29 (95% CI 0.98; 1.69) for germ cell tumours (Table 2, Figure 2A). By contrast, for CNS tumours, analyses yielded worse survival by decreasing AASD (HR_adj_ = 0.93, 95% CI 0.88; 0.98). This pattern was evident for both malignant and non‐malignant CNS tumours.

**TABLE 2:** Adjusted hazard ratios with 95% confidence intervals (CI) of the association between absolute area‐based socioeconomic deprivation (AASD) and 10‐year overall survival from childhood cancer in Germany.

	Hazard ratio (95% CI)^a^, ^b^						
	Total	Female	Male	<1 year	1–4 years	5–9 years	10–14 years
Diagnostic group/cancer type							
All cancer types	1.00 (0.97; 1.03)	0.96 (0.92; 1.00)	1.03 (0.99; 1.07)	1.08 (0.99; 1.18)	1.01 (0.96; 1.06)	0.94 (0.89; 0.99)	1.00 (0.95; 1.06)
Leukaemia	1.03 (0.97; 1.09)	0.98 (0.90; 1.07)	1.07 (0.99; 1.15)	1.15 (0.99; 1.34)	1.07 (0.96; 1.19)	0.98 (0.88; 1.10)	0.98 (0.88; 1.08)
Lymphoblastic leukaemia	0.96 (0.89; 1.03)	0.92 (0.82; 1.03)	0.99 (0.89; 1.09)	1.09 (0.87; 1.37)	1.03 (0.91; 1.17)	0.91 (0.79; 1.05)	0.89 (0.77; 1.01)
Acute myeloid leukaemia	1.10 (0.98; 1.23)	1.02 (0.86; 1.20)	1.18 (1.01; 1.37)	1.27 (0.98; 1.64)	1.15 (0.92; 1.43)	1.12 (0.85; 1.46)	0.97 (0.81; 1.17)
Lymphoma	1.01 (0.88; 1.16)	1.02 (0.81; 1.27)	1.01 (0.84; 1.20)	1.65 (0.73; 3.77)	1.05 (0.76; 1.45)	1.04 (0.80; 1.34)	0.95 (0.78; 1.16)
Hodgkin Lymphoma	1.02 (0.70; 1.49)	0.83 (0.51; 1.36)	1.36 (0.76; 2.41)	n.a.	4.37 (0.67; 28.38)	1.05 (0.50; 2.21)	0.90 (0.57; 1.43)
Non‐Hodgkin Lymphoma	1.07 (0.90; 1.27)	1.08 (0.82; 1.42)	1.06 (0.85; 1.33)	2.49 (0.48; 13.00)	0.98 (0.68; 1.43)	1.09 (0.79; 1.50)	1.07 (0.83; 1.38)
CNS tumours	0.93 (0.88; 0.98)	0.90 (0.84; 0.97)	0.95 (0.88; 1.01)	0.93 (0.80; 1.08)	1.00 (0.91; 1.10)	0.86 (0.79; 0.94)	0.93 (0.85; 1.03)
Malignant	0.95 (0.91; 1.00)	0.94 (0.87; 1.02)	0.96 (0.90; 1.03)	0.91 (0.78; 1.07)	1.03 (0.94; 1.14)	0.91 (0.83; 0.99)	0.94 (0.85; 1.04)
Non‐malignant	0.83 (0.68; 1.02)	0.77 (0.57; 1.03)	0.89 (0.67; 1.18)	0.75 (0.44; 1.28)	0.67 (0.46; 0.96)	0.73 (0.50; 1.09)	1.26 (0.83; 1.90)
Neuroblastoma	1.05 (0.96; 1.15)	0.99 (0.86; 1.14)	1.09 (0.97; 1.23)	1.15 (0.90; 1.48)	1.05 (0.94; 1.17)	0.95 (0.74; 1.24)	0.94 (0.61; 1.47)
Retinoblastoma	0.94 (0.53; 1.68)	0.98 (0.44; 2.18)	0.96 (0.44; 2.09)	0.88 (0.36; 2.16)	1.03 (0.48; 2.22)	n.a.	n.a.
Renal tumours	1.06 (0.88; 1.26)	0.88 (0.69; 1.14)	1.26 (0.98; 1.63)	1.18 (0.81; 1.72)	1.01 (0.78; 1.31)	0.91 (0.64; 1.30)	1.48 (0.78; 2.81)
Hepatic tumours	1.00 (0.79; 1.27)	1.06 (0.71; 1.59)	0.97 (0.73; 1.30)	1.01 (0.52; 1.95)	1.04 (0.74; 1.47)	0.83 (0.48; 1.41)	1.10 (0.63; 1.94)
Bone tumours	0.95 (0.86; 1.06)	0.98 (0.85; 1.14)	0.93 (0.80; 1.08)	1.77 (0.25; 12.45)	0.69 (0.48; 0.99)	0.86 (0.70; 1.06)	1.03 (0.90; 1.17)
Soft tissue sarcomas	0.99 (0.90; 1.09)	0.98 (0.85; 1.12)	1.00 (0.88; 1.13)	0.93 (0.72; 1.21)	0.84 (0.71; 1.00)	0.97 (0.79; 1.20)	1.13 (0.98; 1.30)
Germ cell tumours	1.29 (0.98; 1.69)	0.96 (0.64; 1.44)	1.67 (1.13; 2.45)	1.40 (0.69; 2.85)	1.55 (0.70; 3.42)	2.07 (0.67; 6.38)	1.17 (0.83; 1.66)
Epithelial tumours and melanomas	1.11 (0.86; 1.43)	1.22 (0.84; 1.79)	1.05 (0.74; 1.48)	0.48 (0.03; 7.91)	1.01 (0.57; 1.78)	1.17 (0.64; 2.15)	1.15 (0.83; 1.59)
Other unspecified neoplasms	1.23 (0.54; 2.82)	0.55 (0.16; 1.91)	3.01 (0.44; 20.71)	n.a.	0.54 (0.14; 2.06)	n.a.	n.a.
Diagnostic period^c^							
1997–2001	1.00 (0.96; 1.06)	1.03 (0.95; 1.12)	0.99 (0.93; 1.07)	1.11 (0.94; 1.32)	1.00 (0.91; 1.11)	0.95 (0.86; 1.06)	1.01 (0.92; 1.11)
2002–2006	0.99 (0.94; 1.05)	0.90 (0.83; 0.98)	1.07 (1.00; 1.15)	1.07 (0.91; 1.26)	1.02 (0.93; 1.13)	0.95 (0.84; 1.06)	0.97 (0.88; 1.08)
2007–2011	1.03 (0.97; 1.09)	1.02 (0.93; 1.11)	1.03 (0.95; 1.12)	1.15 (0.95; 1.38)	1.05 (0.93; 1.17)	0.94 (0.83; 1.06)	1.08 (0.97; 1.21)
2012–2016	0.96 (0.89; 1.02)	0.89 (0.80; 0.98)	1.01 (0.93; 1.11)	1.01 (0.83; 1.22)	0.98 (0.87; 1.10)	0.92 (0.81; 1.04)	0.95 (0.84; 1.08)
Birth cohort^d^							
1982–1989	1.00 (0.92; 1.09)	1.12 (0.99; 1.27)	0.91 (0.81; 1.02)			1.05 (0.82; 1.34)	0.99 (0.91; 1.09)
1990–1999	1.01 (0.96; 1.05)	0.94 (0.87; 1.00)	1.07 (1.00; 1.13)	1.13 (0.92; 1.40)	1.03 (0.94; 1.12)	0.95 (0.88; 1.03)	1.02 (0.94; 1.10)
2000–2009	0.98 (0.94; 1.03)	0.95 (0.88; 1.02)	1.02 (0.95; 1.08)	1.09 (0.97; 1.22)	1.02 (0.94; 1.10)	0.92 (0.84; 1.01)	0.97 (0.85; 1.10)
2010–2016	1.00 (0.91; 1.10)	0.91 (0.79; 1.06)	1.07 (0.94; 1.22)	1.04 (0.88; 1.23)	0.96 (0.85; 1.09)	1.15 (0.75; 1.77)	
Place of residence^e^, ^f^							
Urban	0.99 (0.96; 1.02)	0.95 (0.90; 1.00)	1.03 (0.98; 1.07)	1.09 (0.98; 1.20)	1.00 (0.94; 1.06)	0.93 (0.87; 1.00)	0.99 (0.93; 1.05)
Rural	1.02 (0.96; 1.08)	0.99 (0.91; 1.09)	1.03 (0.96; 1.11)	1.06 (0.88; 1.28)	1.04 (0.94; 1.15)	0.95 (0.85; 1.06)	1.04 (0.94; 1.15)

Abbreviations: AASD, absolute area‐based socioeconomic deprivation; CI, confidence interval; CNS, central nervous system.

^a^ Hazard ratios are expressed per 0.3 units (in accordance to the standard deviation/interquartile range of the continuous variable AASD score).

^b^ Adjusted for year of birth, year of diagnosis, place of residence.

^c^ Adjusted for year of birth and place of residence.

^d^ Adjusted for year of diagnosis and place of residence.

^e^ Place of residence was classified as either urban or rural.

^f^ Adjusted for year of birth, year of diagnosis.

Furthermore, we found indications of sex‐ and age‐specific differences. For all cancer types combined, higher AASD appeared to be associated with lower survival (HR_adj_ = 1.03, 95% CI 0.99; 1.07) among males; in females, however, lower AASD appeared to be associated with lower survival (HR_adj_ = 0.96, 95% CI 0.92; 1.00). While we found no evidence for an association between AASD and survival in children aged 1–4 and 10–14 years, infants with higher AASD tended to have worse survival (HR_adj_ = 1.08, 95% CI 0.99; 1.18). Contrarily, children aged 5–9 years with higher AASD had higher survival (HR_adj_ = 0.94, 95% CI 0.89; 0.99) (Table 2).

### 3.3 Subgroup analysis by diagnostic group/cancer type, sex and age group

The association of lower AASD and lower 10‐year OS in CNS tumour patients was observed in both girls and boys; mostly driven by the 5‐ to 9‐year‐olds (HR_adj_ = 0.86, 95% CI 0.79; 0.94) (Table 2, Figures 2 and S6). For some cancer types, we found indications of an association between higher AASD and lower 10‐year OS in males, but not in females. This was the case for AML, neuroblastomas, and germ cell tumours, with HRs_adj_ of 1.18 (95% CI 1.01; 1.37), 1.09 (95% CI 0.97; 1.23) and 1.67 (95% CI 1.13; 2.45) for AASD, respectively. For males diagnosed with HL and renal tumours, we observed tendencies of worse OS with increasing AASD (HR_adj_ = 1.36, 95% CI 0.76; 2.41 and HR_adj_ = 1.26, 95% CI 0.98; 1.63, respectively). By contrast, OS from HL and renal tumours in females appeared to be higher with increasing AASD (HR_adj_ = 0.83, 95% CI 0.51; 1.36 and HR_adj_ = 0.88, 95% CI 0.69; 1.14, respectively) (Table 2, Figure 2B,C). The tendency of higher AASD to associate with lower survival—for AML and neuroblastoma patients in particular—was most pronounced in infants (Table 2, Figure S6).

### 3.4 Single‐indicator SES measures

Contrary to the interpretation of HRs based on the AASD index, a HR below 1 for area‐based household net income indicates that more severe levels of deprivation are associated with lower survival.

**FIGURE 3:**
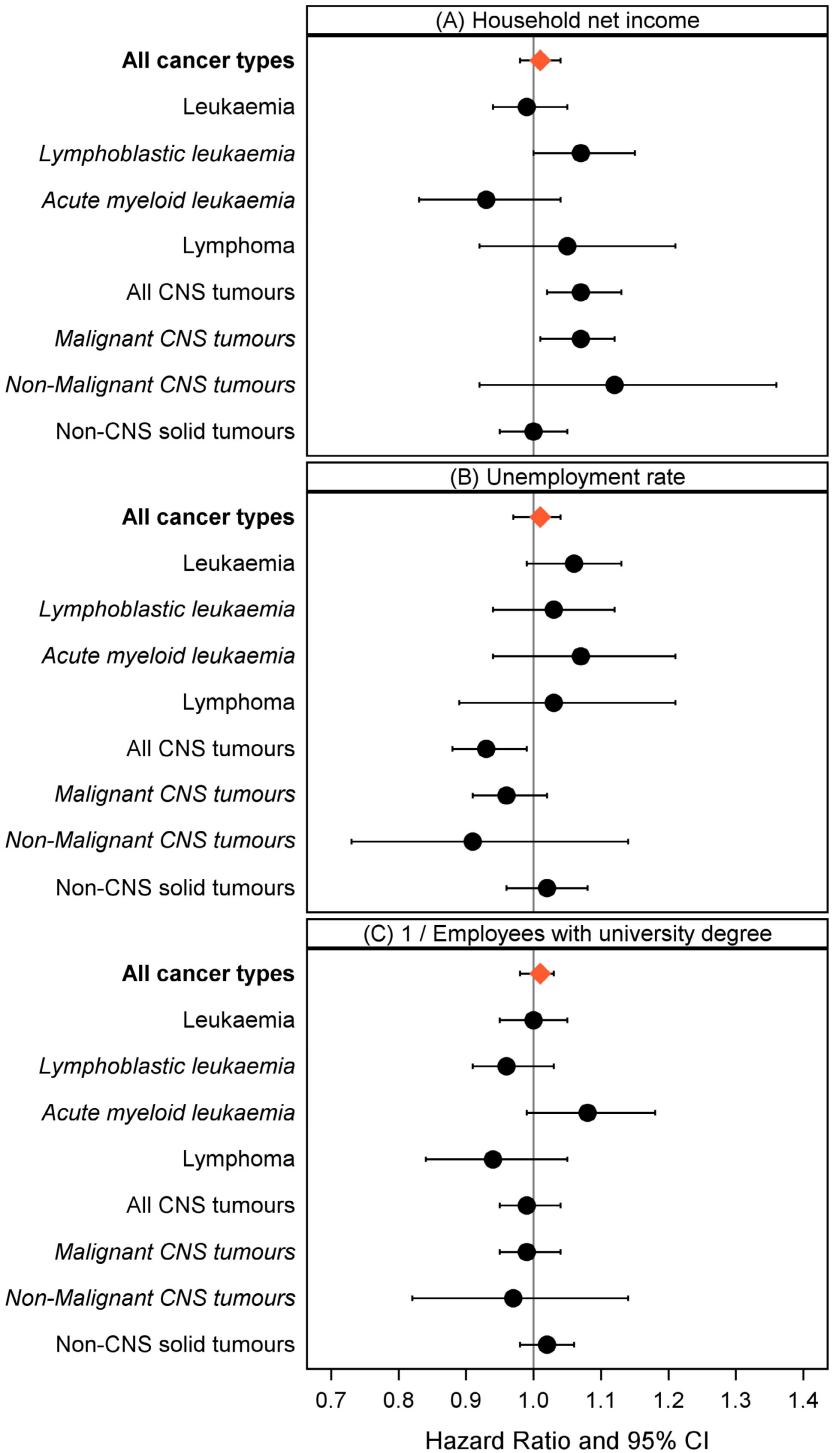
Adjusted hazard ratios of the association between area‐based single indicator SES measures (A) household net income, (B) unemployment rate, and (C) employees with a university degree and childhood cancer survival in Germany. Due to the results of the fractional polynomial regression, the proportions of employees with a university degree have been x^(−1)^ transformed. Hazard ratios are expressed per (A) 250€ household net income, (B) 40 units for unemployment, and (C) 0.05 units for x^(−1)^ transformed employees with a university degree (in accordance with the standard deviation/interquartile range). Hazard ratios were adjusted for year of diagnosis, year of birth, and place of residence (urban/rural). A HR below 1 for area‐based household net income expresses an association of higher deprivation with worse survival. Abbreviations: CI, confidence interval; CNS, central nervous system.

Results for the single indicators household net income, unemployment rate, and proportion of employees with a university degree each revealed null associations with survival from all cancers combined (Figure 3). Nevertheless, lower area‐based household net income appeared to be associated with worse survival from AML (HR_adj_ = 0.93 95% CI 0.83; 1.04), whereas there was an inverse association with survival from lymphoblastic leukaemia (LL) (HR_adj_ = 1.07, 95% CI 1.00; 1.15). Furthermore, we observed an association of lower household net income with higher survival for CNS tumours patients (Figure 3A).

We noted a suggestive association for higher unemployment with worse leukaemia survival (HR_adj_ = 1.06, 95% CI 0.99; 1.13), while for CNS tumor patients, a higher unemployment rate was associated with higher survival (HR_adj_ = 0.93, 95% CI 0.88; 0.99) (Figure 3B).

The association of a higher proportion of employees with a university degree and higher 10‐year OS was evident for AML survival, but not for survival from other tumour types (Figure 3C).

Using the original GISD as well as considering only Western German federal states in additional analyses revealed similar results to those of the main analysis (Tables S2, S3, S4 and S5). In Western German federal states, higher AASD appeared to be associated with lower survival from lymphoblastic leukaemia among 1‐ to 4‐year‐olds. Using the sandwich estimator revealed similar results as the main analysis.

## 4 DISCUSSION

### 4.1 Key findings

With this study, we provide the first assessment of the association between area‐based socioeconomic background and childhood cancer survival in Germany. At first glance, we observed that more severe levels of area‐based socioeconomic deprivation were associated with poorer survival for all cancer diagnoses combined. Adjusting the analyses for year of diagnosis, the association was diminished towards the null. Nonetheless, among children diagnosed with AML and germ cell tumours, lower socioeconomic background appeared to be associated with worse survival. This pattern was most pronounced in male patients and in younger children. Conversely, among children diagnosed with CNS tumours, higher socioeconomic background was associated with worse survival. This pattern was also evident when analysing the single area‐based SES indicators household net income and unemployment rate.

### 4.2 Socioeconomic and healthcare‐related aspects in an international context

When assessing social survival inequalities in childhood cancer, findings should not be generalised to other populations. Instead, results from studies conducted in different countries should be interpreted within their specific context. There are considerable differences in welfare systems, including organisation of and access to healthcare, use of treatment protocols, distance to and coverage of treatment facilities, average socioeconomic level and sociocultural background, as well as methodological differences across studies. For instance, the organisation of healthcare in the United States is markedly different from that of European systems. In the United States, some population groups experience limited healthcare access based on health insurance status, which is often closely linked to SES and employment.^37^

The healthcare and welfare system in Germany has experienced wide‐ranging economic, infrastructural, and societal developments over the past three decades. The former German Democratic Republic (GDR) was characterised by limited financial resources, sparse infrastructure, and poor healthcare provision.^38^, ^39^ Although this region has assimilated to the Western economic system since the German reunification in 1990, an East–West gradient is still present across Germany, with lower average socioeconomic backgrounds in Eastern federal states.^32^, ^38^, ^40^ East–West disparities aside, the socioeconomic heterogeneity in Germany has increased following, for example, multiple waves of immigration from Eastern European countries, Syria and Northern Africa. Immigration has contributed to a more heterogeneous and diverse population, suggesting that social survival inequalities may exist. The findings of the present study did not confirm these suggestions; however, since area‐based SES reflects only one dimension of the complex concept of individual socioeconomic background, social inequalities in childhood cancer may still exist at the individual SES level.

Nevertheless, universal healthcare access is presumed for all families, irrespective of socioeconomic resources or employment, and access to high‐quality healthcare is available with high spatial coverage.^41^ Currently, about 60 paediatric haematology‐oncology units treat children with cancer according to highly standardised treatment protocols for virtually all cancer types.^25^ This highly resourced healthcare system^41^ may have compensated for social inequalities in cancer survival. Moreover, socioeconomic conditions have markedly improved over time (on average), including rising average household net income, increasing levels of educational attainment, and decreasing unemployment rates across the country.^42^ Thus, including the year of diagnosis in the Cox regression models diminished the association between socioeconomic background and childhood cancer survival in Germany, as calendar period can be understood as a surrogate representing multiple factors associated with rising average SES and increasing survival probabilities from treatment advances.^3^, ^42^

### 4.3 Personal socioeconomic factors and cancer survival

Based on the observed social inequalities for childhood cancer survival in numerous European countries with presumed universal access to healthcare, we hypothesised a priori that (parental) socioeconomic characteristics might also affect (the timeliness of) childhood cancer diagnosis, adherence during therapy and follow‐up care, and ultimately, survival in Germany. Consequently, identifying subgroups of children who may benefit from supportive interventions and additional care may contribute to more equitable prognoses for all affected children.

Delays in diagnostics worsen prognosis and elevate the risk of relapse,^10^, ^11^, ^15^ so health literacy and coping behaviour play important roles in navigating the healthcare system, in communication with healthcare providers, and in adherence to treatment recommendations.^5^, ^43^, ^44^, ^45^ Treatment adherence predominantly concerns ALL, which requires a treatment period of more than 2 years.^46^ Lightfoot et al. showed that, for ALL, the SES‐related survival gap widened following the transition to home‐administered treatment, when parents became responsible for drug administration, suggesting poorer treatment adherence in more deprived groups in the UK.^43^ Results from Ireland and Sweden similarly identified this pattern.^11^, ^47^ Contrarily, the present study did not reveal an association between area‐based socioeconomic background and survival from LL. In fact, we found indications of lower household net income being associated with higher survival from LL. In line with this observation, a previous study demonstrated that recent survival probabilities for LL among children from Eastern Germany slightly exceeded those from Western Germany.^48^ Considering the above‐mentioned socioeconomic East–West gradient within Germany, there might be other individual factors influencing treatment adherence.

For the vast majority of childhood cancer types, parental socioeconomic resources (on the area‐based level) apparently did not affect survival in Germany. The dense network of treating hospitals has certainly contributed to ensuring healthcare provision and compensating for any socioeconomic inequalities. Nonetheless, our study revealed some evidence of survival inequalities for certain subgroups, including infants and AML patients. For these patients, treatment is particularly toxic and for parents is likely resource‐intensive. Moreover, as children with AML are more likely to relapse compared to other childhood cancers, the above‐mentioned parental resources (e.g. in terms of adherence to follow‐up care) may be at play in this subgroup. Explanations as to why this pattern was particularly seen in infant males remain largely speculative. Statistical chance cannot be ruled out.

The finding of an inverse association between socioeconomic background and survival from CNS tumours was contrary to our a priori expectation, as research from Switzerland, the UK, and the United States showed that survival disadvantages were most pronounced in paediatric CNS tumour patients with lower SES.^13^, ^49^, ^50^ Moreover, lower survival from CNS tumours was reported to be particularly associated with lower parental education in Switzerland, the Piedmont Region (Italy), Sweden and Finland.^11^, ^15^, ^49^ Since we have no individual information on parental education, our results cannot be related to this possibly influencing factor. We merely observed that—contrary to all other studied SES indicators—the proportion of employees with a university degree was not associated with CNS tumour survival. Finally, the influencing factors for lower survival among CNS tumour patients from areas with higher area‐based socioeconomic background remain unclear.

Notably, our results contrast previous studies on adult cancer survivors that demonstrated an association between socioeconomic status and cancer survival in Germany.^20^, ^22^, ^51^

### 4.4 Strengths and limitations

Our study is strengthened by the high‐quality data from the GCCR, with excellent nationwide coverage of all diagnosed paediatric cancer cases, validated residential address information, and virtually no loss to follow‐up. With the GISD, we used the most suitable area‐based SES measure for public health research in Germany. The availability of socioeconomic information with a geographical resolution on the municipality level enabled data linkage to the cancer patients. Notably, the geographical resolution is lower in some of Germany's larger cities, since there is often only one municipality code reflecting one city. Considering the fact that the socioeconomic heterogeneity is likely higher in urban areas compared with rural areas, our findings may be somewhat biased towards the null. To date, there have been no studies investigating the relationship between the GISD score and individual SES estimates. Since area‐based socioeconomic deprivation reflects only one of multiple dimensions of individual SES, the association between individual SES information and childhood cancer survival may differ from our findings and, further, the inclusion of other SES measures would have significantly complemented our results.

Another strength relates to Germany as the study setting: the universal access to healthcare in Germany irrespective of socioeconomic conditions, and the sizeable childhood population of about 11 million, which enabled comprehensive subgroup analyses with high statistical power. However, a limitation is the lack of information about disease stage and grade at diagnosis. Since it is well established that lower socioeconomic status is associated with advanced disease^10^, ^15^ and that timely diagnosis is essential for good prognosis, analyses by stage may have given further important insights.

## 5 CONCLUSION

In summary, we found little evidence for social inequalities in childhood cancer survival in Germany. However, since our analysis is based on area‐based SES, which may not directly reflect the individual SES of a family, the presence of social inequalities with respect to other dimensions of SES cannot be ruled out. Therefore, assessing survival inequalities with individual SES information may shed light on the survival patterns among children with cancer in Germany more precisely. Moreover, with the aim of achieving higher cure rates and equity for every affected child, further research on potential underlying mechanisms driving survival inequalities, such as migration background, is warranted.

## AUTHOR CONTRIBUTIONS

**Maike Wellbrock:** Conceptualization; methodology; formal analysis; data curation; writing – original draft; writing – review and editing; visualization; project administration; funding acquisition. **Arndt Borkhardt:** Conceptualization; writing – review and editing. **Cécile M. Ronckers:** Data curation; writing – review and editing. **Claudia Spix:** Data curation; writing – review and editing. **Desiree Grabow:** Data curation; writing – review and editing. **Anna‐Liesa Filbert:** Writing – review and editing. **Daniel Wollschläger:** Conceptualization; methodology; formal analysis; data curation; writing – review and editing; supervision; project administration. **Friederike Erdmann:** Conceptualization; methodology; data curation; supervision; funding acquisition; project administration; writing – review and editing.

## FUNDING INFORMATION

This research was funded by a grant from the *Tour der Hoffnung* foundation. The *Tour der Hoffnung* foundation was not involved in the conceptualisation, design, content or preparation of the manuscript, nor the decision to submit for publication.

## CONFLICT OF INTEREST STATEMENT

The authors declare that the research was conducted in the absence of any commercial, personal or financial relationships with other people or organisations that could be construed as a potential conflict of interest.

## ETHICS STATEMENT

No ethics approval or consent was required for this study. This research was carried out in accordance with the Code of Ethics of the World Medical Association (Declaration of Helsinki) for experiments involving humans.

## Supporting information


**Appendix S1:** Supporting information.

## Data Availability

The data that support the findings of this study are available from the German Childhood Cancer Registry (as aggregated data) upon reasonable request and in compliance with national data protection regulations. Further information is available from the corresponding author upon request.
