# Supplementary material for: Socioeconomic background and childhood cancer survival in Germany: A nationwide assessment based on data from the German Childhood Cancer Registry
Source: Int J Cancer. 2025 Jul 17;157(11):2235–47. doi: 10.1002/ijc.70042 (PMC12496003; doi:10.1002/ijc.70042)
Supplement: Supplementary file 1 — Appendix S1: Supporting information. [file IJC-157-2235-s001.pdf]

Supplementary material

Socioeconomic background and childhood cancer survival in Germany – A nationwide assessment based on data from the German Childhood Cancer Registry

Maike Wellbrock, Arndt Borkhardt, Cecile Ronckers, Claudia Spix, Desiree Grabow, Anna-Liesa Filbert, Daniel Wollschläger, Friederike Erdmann

Table of content

	Page
Figure S1: Flow chart reflecting the development of the analytical sample following exclusion criteria	1
Figure S2: Directed acyclic graph (DAG) reflecting theoretical considerations about causal relationships for socioeconomic background and childhood cancer survival	2
Figure S3: Distribution of (A) absolute area-based socioeconomic deprivation scores, (B) German Index of Socioeconomic Deprivation scores, (C) household net income, (D) unemployment and (E) employees with a university degree among children with cancer diagnosed at ages 0-14 years between 1997 and 2016 in Germany	3
Figure S4: Contribution of the dimensions income, occupation and education to the absolute area-based socioeconomic deprivation (AASD) measure in Germany over time (arithmetic mean of values)	4
Figure S5: Temporal development of the arithmetic mean of absolute area-based socioeconomic deprivation (AASD) in children with cancer diagnosed at ages 0-14 years between 1997 and 2016 compared to the German average	5
Figure S6: Adjusted hazard ratios with 95% confidence intervals (CI) for the association between area-based socioeconomic deprivation (AASD) and 10-year overall survival from childhood cancer in Germany (1997-2016) for (A) children aged <1 year at diagnosis, (B) children aged 1-4 years at diagnosis, (C) children aged 5-9 years at diagnosis and (D) children aged 10-14 years at diagnosis.	6
Table S1: Crude hazard ratios (HR) with 95% confidence intervals (CI) of the association between absolute area-based socioeconomic deprivation (AASD) and 10-year overall survival from childhood cancer in Germany	8
Table S2: Crude hazard ratios with 95% confidence intervals (CI) of the association between area-based socioeconomic deprivation and 10-year overall survival from childhood cancer in Germany using the German Index of Socioeconomic Deprivation	10
Table S3: Adjusted hazard ratios with 95% confidence intervals (CI) of the association between area-based socioeconomic deprivation and 10-year overall survival from childhood cancer in Germany using the German Index of Socioeconomic Deprivation	12

Table S4: Adjusted hazard ratios with 95% confidence intervals (CI) of the association between absolute area-based socioeconomic deprivation (AASD) and 10-year overall survival from childhood cancer in Germany: additional analysis (Western German federal states only)	14
Table S5: Adjusted hazard ratios with 95% confidence intervals (CI) of the association between single SES indicators and 10-year overall survival from childhood cancer in Germany: additional analysis (Western German federal states only)	16
Removing the normalization step from the original GISD<br>Figure S7: Scatterplot of the AASD against the GISD with the colour-wash indicating the calendar year. Each point represents one municipality in a separate year.	17

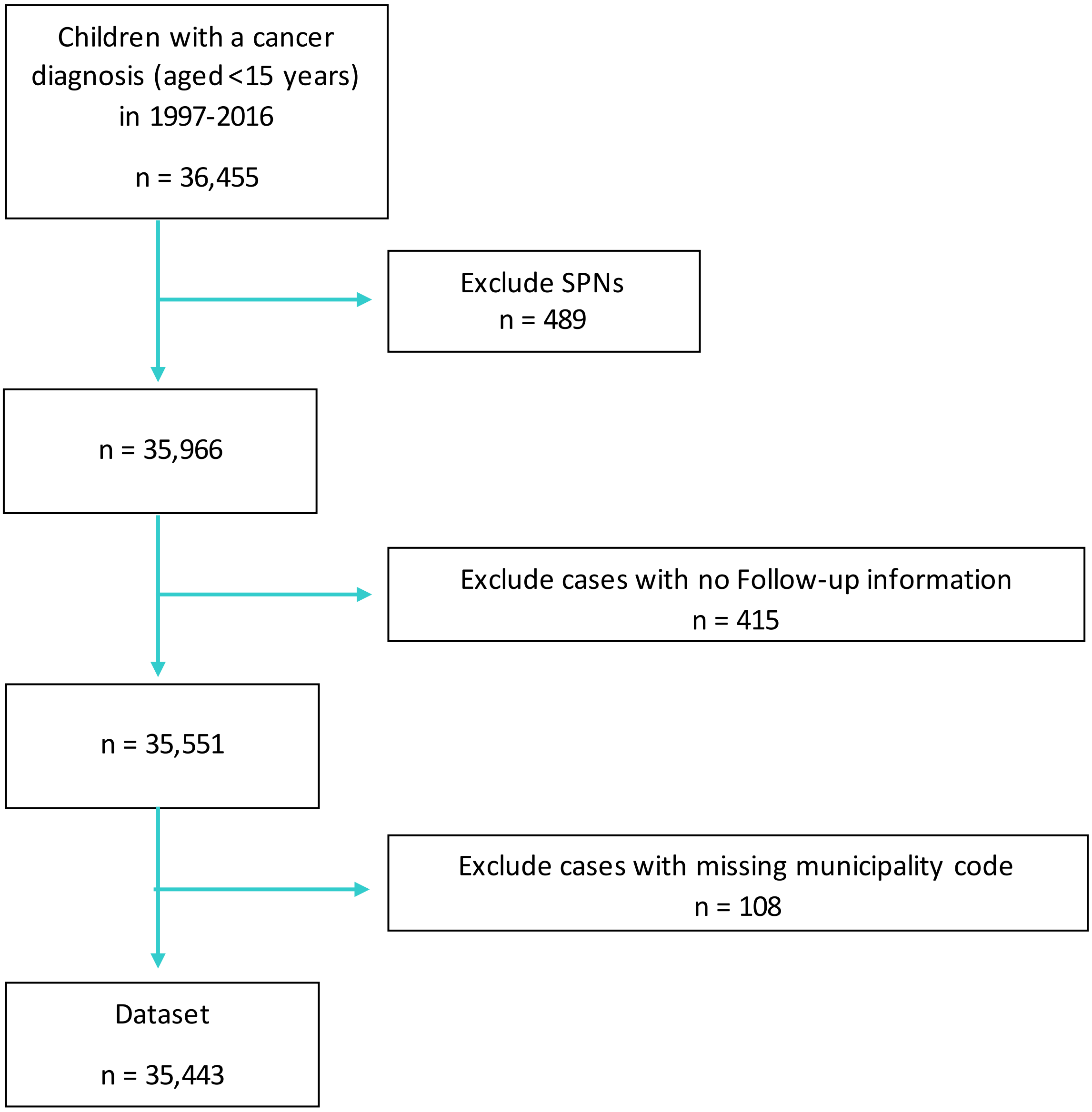

Figure S1: Flow chart of the analytical sample after applying the inclusion and exclusion criteria
Abbreviations: n, number; SPN, subsequent primary neoplasm

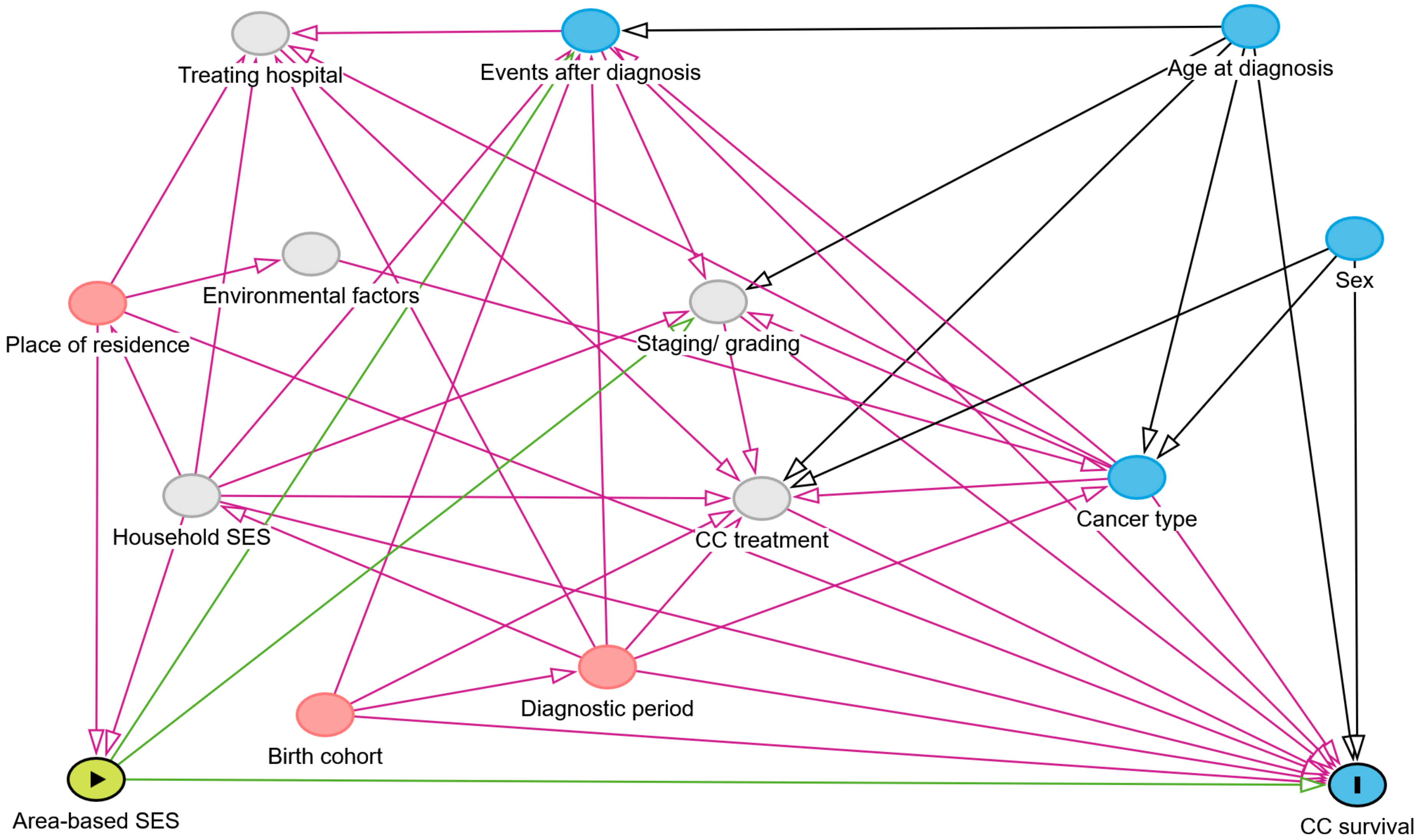

Figure S2: Directed acyclic graph (DAG) reflecting theoretical considerations about causal relationships for socioeconomic background and childhood cancer survival. Area-based SES (socioeconomic status) represents the exposure variable (green), CC (childhood cancer) survival represents the outcome variable (blue), confounders are displayed in red, other measured covariates are displayed in blue and unmeasured/ unknown factors are displayed in grey.

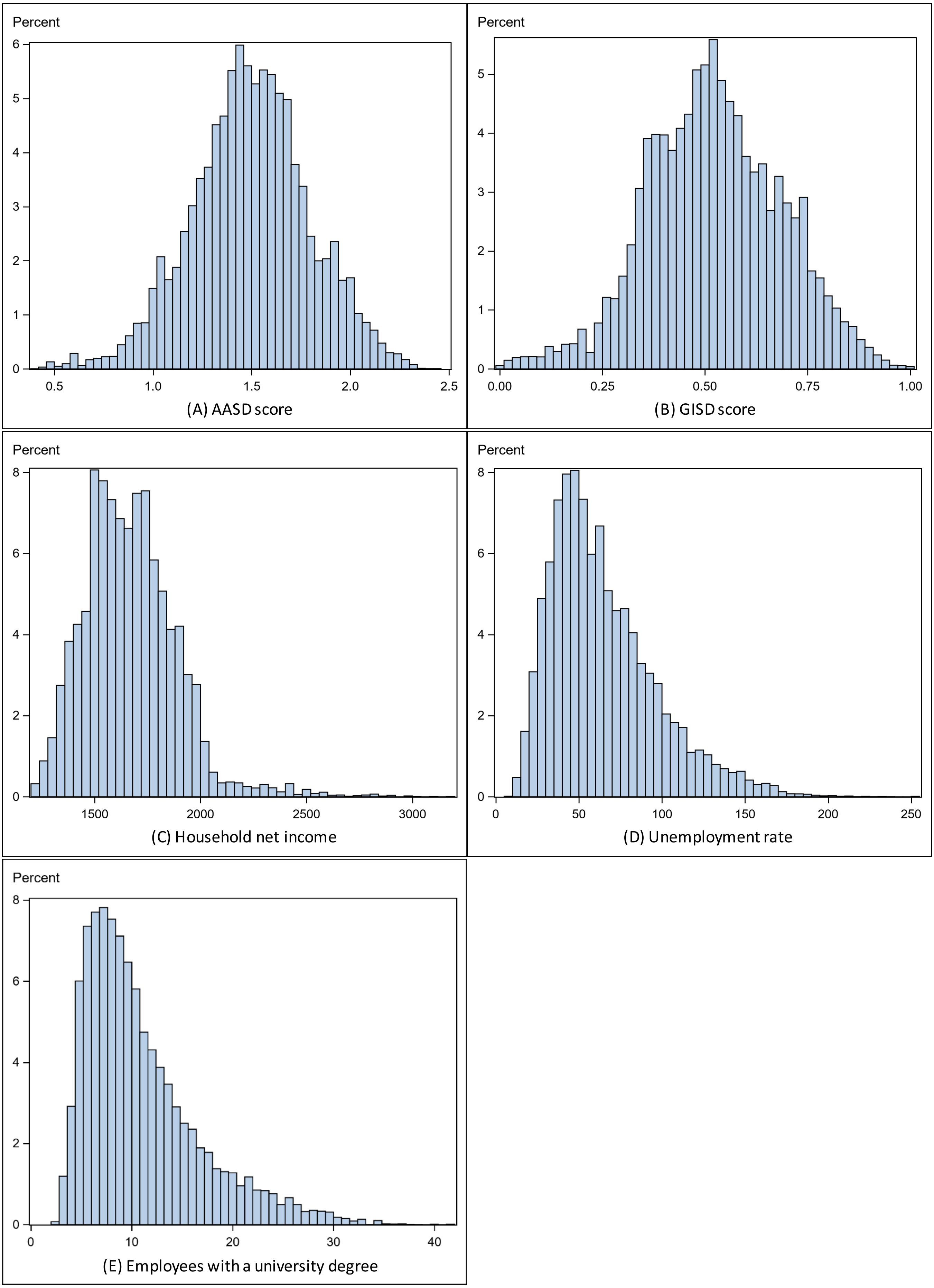

Figure S3: Distribution of all exposure variables in the study population: (A) absolute area-based socioeconomic deprivation scores, (B) German Index of Socioeconomic Deprivation scores, (C) household net income, (D) unemployment and (E) employees with a university degree

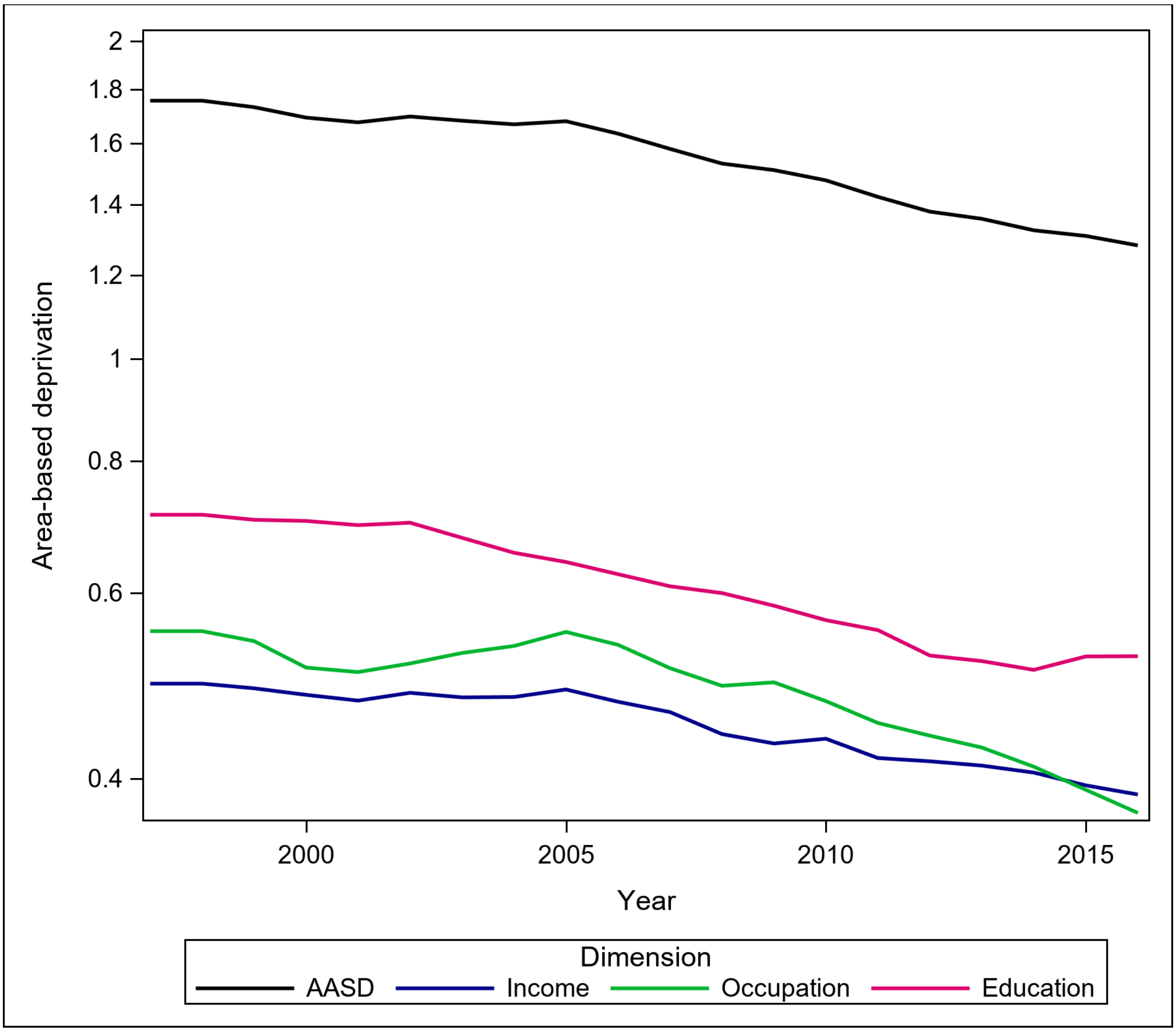

Figure S4: Contribution of the dimensions income, occupation and education to the absolute area-based socioeconomic deprivation (AASD) measure in Germany over time (arithmetic mean of values)

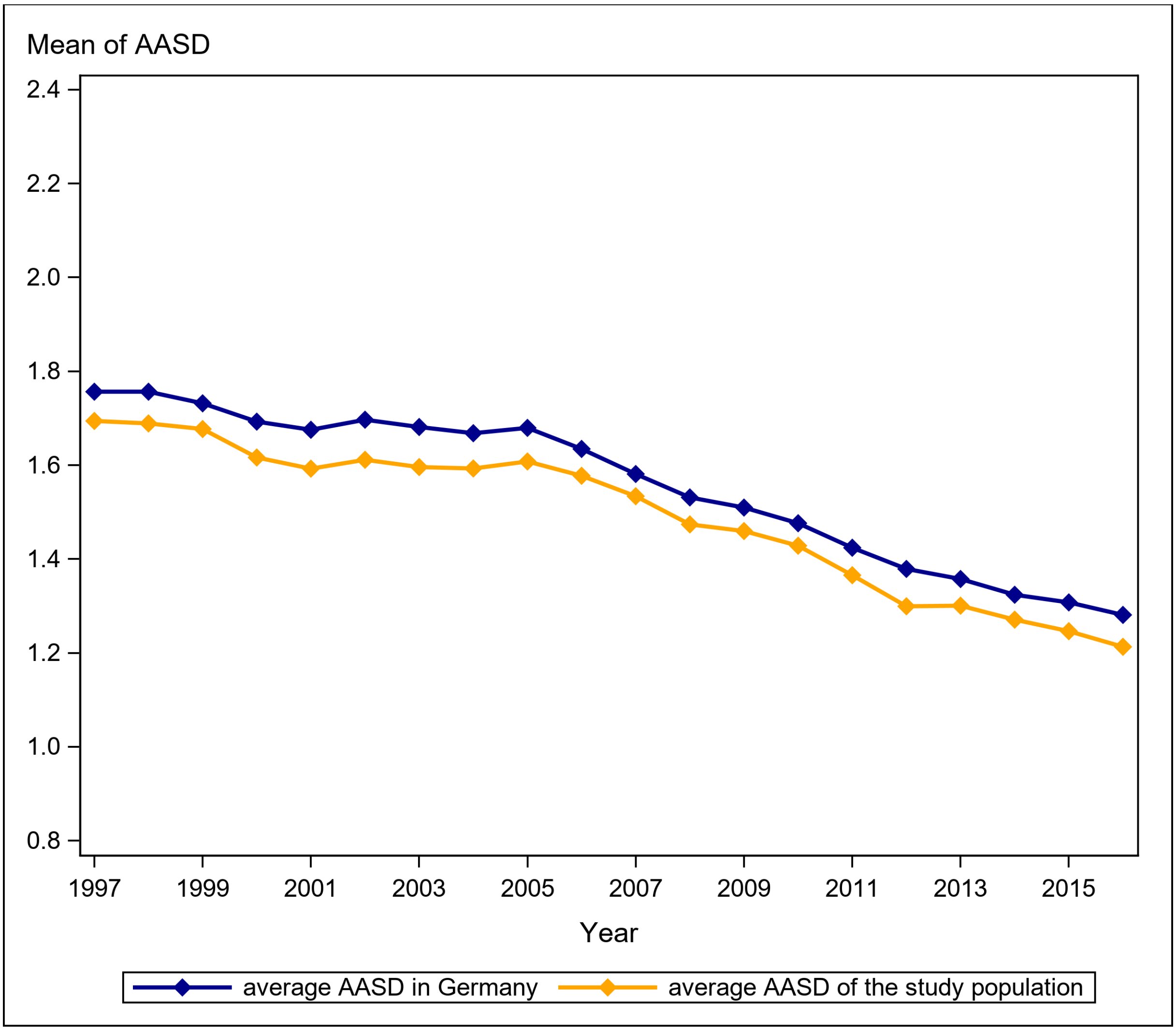

Figure S5: Temporal development of the arithmetic mean of absolute area-based socioeconomic deprivation (AASD) in children with cancer diagnosed at ages 0-14 years between 1997 and 2016 compared to the German average

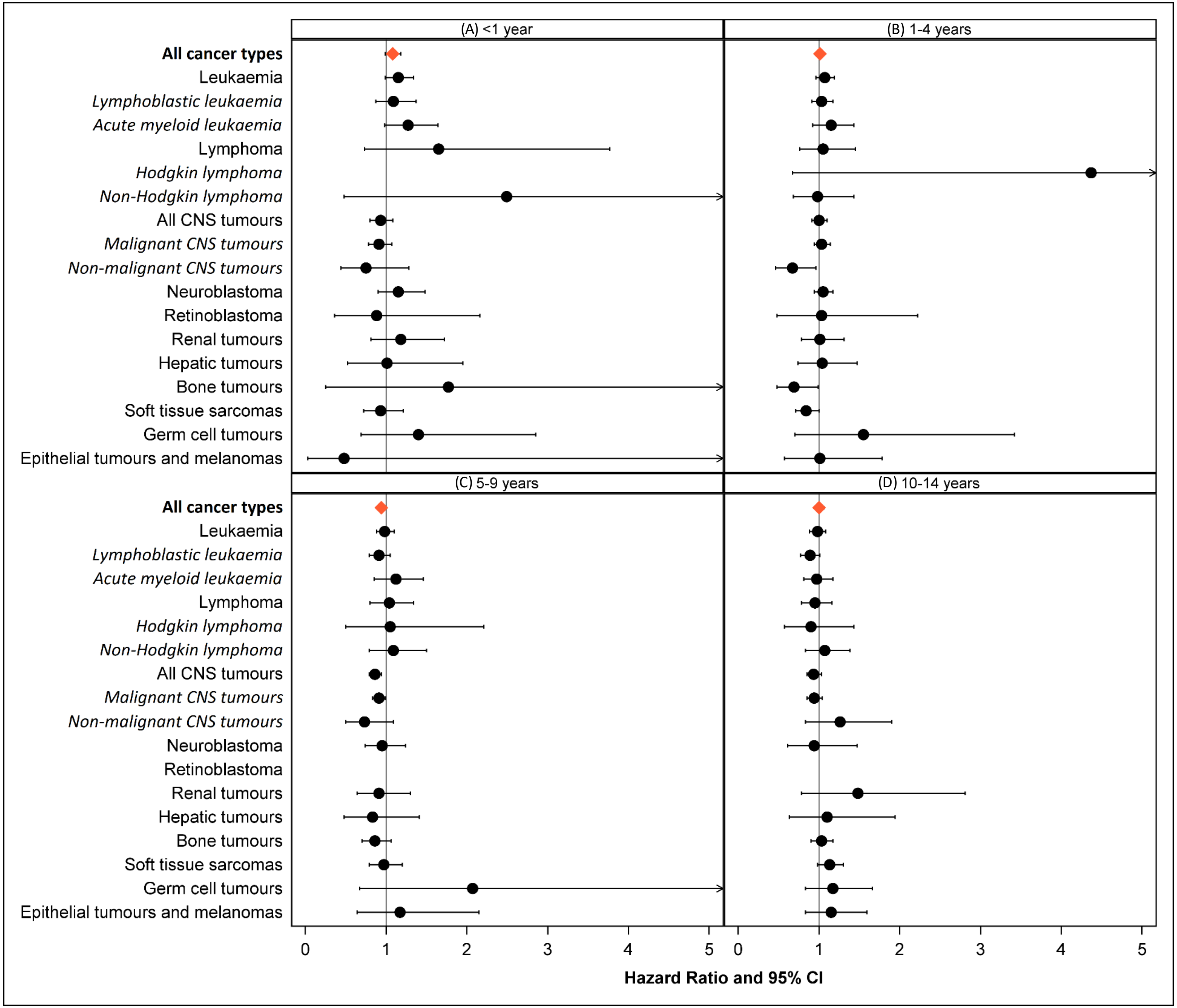

(A) <1 year
(B) 1-4 years
(C) 5-9 years
(D) 10-14 years
All cancer types
Leukaemia
Lymphoblastic leukaemia
Acute myeloid leukaemia
Lymphoma
Hodgkin lymphoma
Non-Hodgkin lymphoma
All CNS tumours
Malignant CNS tumours
Non-malignant CNS tumours
Neuroblastoma
Retinoblastoma
Renal tumours
Hepatic tumours
Bone tumours
Soft tissue sarcomas
Germ cell tumours
Epithelial tumours and melanomas
0
1
2
3
4
5
Hazard Ratio and 95% CI

Figure S6: Adjusted hazard ratios with 95% confidence intervals (CI) for the association between area-based socioeconomic deprivation (AASD) and 10-year overall survival from childhood cancer in Germany (1997-2016) for (A) children aged <1 year at diagnosis, (B) children aged 1-4 years at diagnosis, (C) children aged 5-9 years at diagnosis and (D) children aged 10-14 years at diagnosis.
Hazard ratios are expressed per 0.3 units AASD in accordance with the standard deviation/ interquartile range. Hazard ratios were adjusted for year of diagnosis and place of residence (urban/ rural).
Abbreviations: CI, confidence interval, CNS; central nervous system

Table S1: Crude hazard ratios (HR) with 95% confidence intervals (CI) of the univariable association between absolute area-based socioeconomic deprivation (AASD) and 10-year overall survival from childhood cancer in Germany

	Hazard ratio (95% CI)[a]						
	Total	Female	Male	<1 year	1-4 years	5-9 years	10-14 years
Diagnostic group/ cancer type							
All cancer types	1.07 (1.05; 1.10)	1.03 (0.99; 1.07)	1.11 (1.07; 1.14)	1.13 (1.05; 1.22)	1.07 (1.02; 1.12)	1.02 (0.97; 1.07)	1.10 (1.05; 1.15)
Leukaemia	1.18 (1.12; 1.24)	1.13 (1.04; 1.22)	1.23 (1.15; 1.31)	1.25 (1.10; 1.44)	1.17 (1.07; 1.29)	1.15 (1.04; 1.27)	1.18 (1.08; 1.29)
Lymphoblastic leukaemia	1.09 (1.02; 1.16)	1.04 (0.94; 1.15)	1.13 (1.04; 1.23)	1.11 (0.91; 1.35)	1.14 (1.02; 1.28)	1.05 (0.93; 1.19)	1.07 (0.95; 1.20)
Acute myeloid leukaemia	1.27 (1.15; 1.40)	1.17 (1.02; 1.35)	1.37 (1.19; 1.57)	1.42 (1.12; 1.79)	1.27 (1.05; 1.53)	1.36 (1.08; 1.71)	1.12 (0.95; 1.32)
Lymphoma	1.14 (1.01; 1.28)	1.10 (0.91; 1.34)	1.16 (0.99; 1.34)	1.58 (0.86; 2.92)	1.28 (0.97; 1.68)	1.20 (0.97; 1.50)	1.04 (0.88; 1.24)
Hodgkin Lymphoma	1.26 (0.91; 1.75)	1.06 (0.69; 1.62)	1.54 (0.94; 2.54)	n.a.	3.84 (1.03; 14.45)	1.43 (0.75; 2.72)	1.09 (0.74; 1.62)
Non-Hodgkin Lymphoma	1.22 (1.05; 1.41)	1.14 (0.90; 1.45)	1.25 (1.03; 1.52)	2.17 (0.58; 8.10)	1.22 (0.88; 1.69)	1.27 (0.97; 1.67)	1.18 (0.95; 1.47)
CNS tumours	1.01 (0.97; 1.06)	0.96 (0.90; 1.03)	1.05 (0.99; 1.11)	1.02 (0.89; 1.17)	1.09 (1.01; 1.18)	0.93 (0.86; 1.00)	1.05 (0.97; 1.14)
Malignant	1.03 (0.98; 1.07)	0.99 (0.93; 1.07)	1.05 (0.99; 1.11)	0.97 (0.84; 1.12)	1.13 (1.04; 1.23)	0.96 (0.89; 1.04)	1.01 (0.93; 1.10)
Non-malignant	1.03 (0.87; 1.23)	0.93 (0.72; 1.20)	1.13 (0.89; 1.43)	1.20 (0.80; 1.81)	0.81 (0.58; 1.12)	0.90 (0.65; 1.26)	1.52 (1.08; 2.14)
Neuroblastoma	1.06 (0.98; 1.15)	1.03 (0.90; 1.17)	1.09 (0.98; 1.21)	1.15 (0.92; 1.43)	1.02 (0.93; 1.13)	0.94 (0.75; 1.17)	1.05 (0.75; 1.49)
Retinoblastoma	1.09 (0.67; 1.79)	1.00 (0.51; 1.97)	1.21 (0.59; 2.49)	0.84 (0.40; 1.76)	1.32 (0.67; 2.59)	n.a.	n.a.
Renal tumours	1.11 (0.95; 1.30)	0.96 (0.77; 1.19)	1.30 (1.04; 1.63)	1.45 (0.06; 1.99)	1.02 (0.81; 1.29)	0.92 (0.67; 1.26)	1.56 (0.87; 2.81)
Hepatic tumours	1.23 (1.01; 1.50)	1.43 (1.02; 2.02)	1.15 (0.90; 1.46)	1.39 (0.79; 2.44)	1.19 (0.88; 1.62)	1.19 (0.78; 1.81)	1.09 (0.70; 1.68)
Bone tumours	0.98 (0.89; 1.07)	0.99 (0.87; 1.12)	1.00 (0.85; 1.10)	1.48 (0.36; 6.14)	0.76 (0.56; 1.02)	0.97 (0.81; 1.16)	1.01 (0.91; 1.13)
Soft tissue sarcomas	1.02 (0.94; 1.11)	1.04 (0.93; 1.18)	1.00 (0.90; 1.12)	0.96 (0.76; 1.21)	0.88 (0.75; 1.02)	1.00 (0.84; 1.20)	1.16 (1.02; 1.32)

Germ cell tumours	1.16 (0.92; 1.47)	1.04 (0.74; 1.46)	1.27 (0.91; 1.77)	1.04 (0.58; 1.86)	1.20 (0.58; 2.47)	2.01 (0.87; 4.62)	1.10 (0.83; 1.46)
Epithelial tumours and melanomas	1.31 (1.05; 1.63)	1.33 (0.96; 1.84)	1.34 (0.99; 1.84)	n.a.	1.06 (0.62; 1.80)	1.50 (0.89; 2.50)	1.34 (1.02; 1.78)
Other unspecified neoplasms	1.11 (0.60; 2.05)	0.74 (0.32; 1.72)	1.91 (0.79; 4.60)	n.a.	0.78 (0.29; 2.10)	1.93 (0.49; 7.56)	1.23 (0.33; 4.58)
Diagnostic period							
1997-2001	1.03 (0.97; 1.08)	1.04 (0.96; 1.12)	1.01 (0.94; 1.09)	1.12 (0.95; 1.33)	1.00 (0.91; 1.11)	0.99 (0.89; 1.10)	1.02 (0.93; 1.12)
2002-2006	1.00 (0.95; 1.05)	0.91 (0.83; 0.99)	1.07 (1.00; 1.15)	1.09 (0.93; 1.28)	1.02 (0.93; 1.13)	0.94 (0.84; 1.05)	0.97 (0.88; 1.07)
2007-2011	1.04 (0.98; 1.10)	1.02 (0.94; 1.12)	1.04 (0.96; 1.13)	1.14 (0.95; 1.37)	1.06 (0.95; 1.18)	0.94 (0.84; 1.06)	1.07 (0.96; 1.19)
2012-2016	0.96 (0.90; 1.03)	0.90 (0.80; 0.99)	1.02 (0.94; 1.11)	1.01 (0.84; 1.22)	0.98 (0.88; 1.10)	0.93 (0.82; 1.05)	0.96 (0.85; 1.09)
Birth cohort							
1982-1989	1.01 (0.93; 1.10)	1.11 (0.99; 1.26)	0.94 (0.84; 1.04)			1.03 (0.81; 1.31)	1.01 (0.92; 1.10)
1990-1999	1.02 (0.98; 1.07)	0.95 (0.89; 1.02)	1.08 (1.02; 1.14)	1.15 (0.93; 1.41)	1.03 (0.94; 1.12)	0.97 (0.90; 1.04)	1.06 (0.99; 1.14)
2000-2009	1.03 (0.99; 1.08)	0.99 (0.93; 1.06)	1.07 (1.01; 1.13)	1.12 (0.99; 1.25)	1.07 (1.00; 1.15)	0.92 (0.84; 1.00)	0.99 (0.87; 1.13)
2010-2016	1.00 (0.91; 1.10)	0.92 (0.80; 1.07)	1.07 (0.94; 1.21)	1.03 (0.88; 1.21)	0.97 (0.86; 1.10)	1.09 (0.72; 1.63)	
Place of residence							
Urban	1.06 (1.03; 1.09)	1.01 (0.97; 1.06)	1.09 (1.05; 1.14)	1.14 (1.04; 1.24)	1.05 (0.99; 1.11)	1.01 (0.95; 1.07)	1.07 (1.02; 1.13)
Rural	1.11 (1.06; 1.16)	1.08 (1.00; 1.16)	1.13 (1.06; 1.21)	1.09 (0.93; 1.28)	1.11 (1.02; 1.21)	1.06 (0.97; 1.17)	1.16 (1.06; 1.26)

[a]Hazard ratios > 1 indicate that a higher mortality rate was associated with higher deprivation

[b]HRs are expressed per 0.3 units (in accordance to the standard deviation and interquartile range of the continuous variable AASD score)

Abbreviations: CI, confidence interval; CNS, central nervous system; n.a., not applicable (due to small numbers)

Table S2: Crude hazard ratios with 95% confidence intervals (CI) of the univariable association between area-based socioeconomic deprivation and 10-year overall survival from childhood cancer in Germany using the German Index of Socioeconomic Deprivation

	Crude Hazard ratio (95% CI)[a,b]						
	Total	Female	Male	<1 year	1-4 years	5-9 years	10-14 years
Diagnostic group/ cancer type							
All cancer types	1.00 (0.97; 1.03)	0.96 (0.92; 1.01)	1.03 (0.99; 1.07)	1.09 (0.99; 1.19)	1.00 (0.95; 1.06)	0.94 (0.89; 1.00)	1.00 (0.95; 1.06)
Leukaemia	1.05 (0.98; 1.11)	1.00 (0.92; 1.10)	1.08 (1.00; 1.17)	1.17 (1.00; 1.37)	1.06 (0.95; 1.18)	0.99 (0.88; 1.12)	1.02 (0.92; 1.14)
Lymphoblastic leukaemia	0.97 (0.90; 1.04)	0.94 (0.83; 1.06)	0.99 (0.90; 1.09)	1.07 (0.83; 1.36)	1.01 (0.89; 1.16)	0.92 (0.80; 1.07)	0.92 (0.80; 1.06)
Acute myeloid leukaemia	1.12 (1.00; 1.26)	1.06 (0.89; 1.26)	1.18 (1.00; 1.38)	1.38 (1.04; 1.83)	1.14 (0.91; 1.43)	1.10 (0.84; 1.44)	0.97 (0.80; 1.18)
Lymphoma	1.00 (0.87; 1.15)	0.97 (0.77; 1.23)	1.02 (0.85; 1.22)	1.35 (0.64; 2.86)	1.08 (0.77; 1.51)	1.02 (0.79; 1.32)	0.97 (0.78; 1.17)
Hodgkin Lymphoma	1.03 (0.70; 1.52)	0.77 (0.46; 1.29)	1.45 (0.81; 2.62)	n.a.	4.10 (0.73; 22.96)	1.18 (0.56; 2.48)	0.87 (0.54; 1.40)
Non-Hodgkin Lymphoma	1.08 (0.90; 1.29)	1.08 (0.81; 1.44)	1.07 (0.85; 1.35)	1.77 (0.46; 6.80)	1.04 (0.70; 1.56)	1.09 (0.79; 1.51)	1.08 (0.83; 1.41)
CNS tumours	0.92 (0.87; 0.97)	0.90 (0.83; 0.98)	0.93 (0.87; 1.00)	0.92 (0.79; 1.08)	0.99 (0.90; 1.09)	0.86 (0.78; 0.94)	0.92 (0.83; 1.03)
Malignant	0.95 (0.90; 1.00)	0.94 (0.87; 1.02)	0.95 (0.89; 1.02)	0.90 (0.77; 1.07)	1.03 (0.93; 1.13)	0.91 (0.83; 1.00)	0.92 (0.82; 1.02)
Non-malignant	0.80 (0.64; 0.99)	0.72 (0.53; 0.98)	0.87 (0.65; 1.17)	0.77 (0.47; 1.27)	0.66 (0.45; 0.96)	0.75 (0.50; 1.13)	1.32 (0.86; 2.03)
Neuroblastoma	1.07 (0.98; 1.18)	0.98 (0.85; 1.14)	1.14 (1.01; 1.29)	1.17 (0.90; 1.52)	1.04 (0.93; 1.17)	0.95 (0.73; 1.23)	0.98 (0.62; 1.53)
Retinoblastoma	0.96 (0.53; 1.73)	1.00 (0.43; 2.32)	0.92 (0.40; 2.12)	0.80 (0.32; 1.98)	1.06 (0.48; 2.34)	n.a.	n.a.
Renal tumours	1.10 (0.91; 1.32)	0.90 (0.69; 1.17)	1.36 (1.04; 1.79)	1.35 (0.92; 1.98)	1.04 (0.79; 1.36)	0.97 (0.65; 1.43)	1.38 (0.67; 2.87)
Hepatic tumours	0.94 (0.75; 1.19)	1.07 (0.72; 1.58)	0.88 (0.66; 1.17)	0.93 (0.50; 1.72)	0.92 (0.65; 1.31)	0.82 (0.49; 1.40)	1.11 (0.63; 1.96)
Bone tumours	0.93 (0.84; 1.04)	0.96 (0.82; 1.12)	0.91 (0.78; 1.06)	1.38 (0.33; 5.84)	0.68 (0.46; 1.02)	0.85 (0.68; 1.06)	1.00 (0.87; 1.14)
Soft tissue sarcomas	0.99 (0.90; 1.09)	0.96 (0.83; 1.11)	1.02 (0.89; 1.16)	0.96 (0.73; 1.26)	0.80 (0.67; 0.96)	0.97 (0.79; 1.21)	1.16 (0.99; 1.35)

Germ cell tumours	1.33 (1.00; 1.77)	0.93 (0.62; 1.39)	1.87 (1.25; 2.81)	1.38 (0.67; 2.84)	1.59 (0.69; 3.71)	2.03 (0.75; 5.47)	1.14 (0.80; 1.63)
Epithelial tumours and melanomas	1.04 (0.80; 1.35)	1.11 (0.75; 1.64)	1.00 (0.70; 1.44)	0.76 (0.17; 3.39)	0.99 (0.56; 1.74)	1.16 (0.63; 2.13)	1.07 (0.76; 1.50)
Other unspecified neoplasms	1.41 (0.67; 2.99)	0.69 (0.20; 2.33)	2.66 (0.88; 8.08)	n.a.	0.51 (0.15; 1.75)	n.a.	2.13 (0.54; 9.96)
Diagnostic period							
1997-2001	1.02 (0.97; 1.08)	1.04 (0.96; 1.13)	1.01 (0.94; 1.08)	1.12 (0.95; 1.32)	1.00 (0.91; 1.10)	0.98 (0.89; 1.09)	1.02 (0.93; 1.12)
2002-2006	1.00 (0.94; 1.06)	0.90 (0.82; 0.99)	1.08 (1.00; 1.17)	1.09 (0.92; 1.30)	1.03 (0.92; 1.14)	0.94 (0.83; 1.06)	0.97 (0.87; 1.08)
2007-2011	1.03 (0.97; 1.10)	1.01 (0.91; 1.12)	1.05 (0.96; 1.15)	1.16 (0.95; 1.42)	1.05 (0.93; 1.18)	0.95 (0.83; 1.08)	1.06 (0.94; 1.19)
2012-2016	0.97 (0.90; 1.03)	0.89 (0.81; 0.99)	1.03 (0.93; 1.13)	1.01 (0.83; 1.24)	0.99 (0.88; 1.12)	0.93 (0.81; 1.06)	0.96 (0.84; 1.09)
Birth cohort							
1982-1989	1.01 (0.93; 1.10)	1.12 (0.99; 1.26)	0.93 (0.83; 1.04)			1.03 (0.82; 1.31)	1.01 (0.92; 1.10)
1990-1999	1.00 (0.96; 1.05)	0.93 (0.87; 1.00)	1.07 (1.00; 1.14)	1.14 (0.93; 1.39)	1.03 (0.94; 1.12)	0.95 (0.88; 1.03)	1.01 (0.93; 1.09)
2000-2009	0.98 (0.93; 1.03)	0.95 (0.88; 1.02)	1.01 (0.94; 1.08)	1.09 (0.96; 1.24)	1.00 (0.93; 1.09)	0.92 (0.83; 1.01)	0.97 (0.84; 1.11)
2010-2016	1.00 (0.91; 1.11)	0.91 (0.78; 1.06)	1.08 (0.94; 1.24)	1.05 (0.88; 1.25)	0.97 (0.85; 1.12)	1.10 (0.71; 1.69)	
Place of residence							
Urban	0.99 (0.95; 1.02)	0.94 (0.89; 0.99)	1.02 (0.98; 1.07)	1.08 (0.97; 1.20)	0.98 (0.92; 1.05)	0.94 (0.87; 1.01)	0.99 (0.93; 1.06)
Rural	1.02 (0.96; 1.09)	1.00 (0.91; 1.10)	1.04 (0.96; 1.12)	1.07 (0.88; 1.30)	1.04 (0.93; 1.16)	0.96 (0.86; 1.08)	1.05 (0.94; 1.16)

[a]Hazard ratios > 1 indicate that a higher mortality rate was associated with higher deprivation.

[b]HRs are expressed per 0.2 units (in accordance to the interquartile range/ standard deviation of the continuous variable GISD score)

Abbreviations: CI, confidence interval; CNS, central nervous system; n.a., not applicable (due to small numbers)

Table S3: Adjusted hazard ratios with 95% confidence intervals (CI) of the association between area-based socioeconomic deprivation and 10-year overall survival from childhood cancer in Germany using the German Index of Socioeconomic Deprivation

	Hazard ratio (95% CI)[a,b,c]						
	Total	Female	Male	<1 year	1-4 years	5-9 years	10-14 years
Diagnostic group/ cancer type							
All cancer types	1.00 (0.97; 1.03)	0.96 (0.92; 1.01)	1.03 (0.99; 1.07)	1.08 (0.99; 1.19)	1.01 (0.95; 1.06)	0.95 (0.89; 1.01)	1.00 (0.95; 1.06)
Leukaemia	1.04 (0.98; 1.11)	1.00 (0.91; 1.09)	1.08 (1.00; 1.17)	1.15 (0.98; 1.35)	1.06 (0.95; 1.19)	1.01 (0.89; 1.13)	1.02 (0.91; 1.13)
Lymphoblastic leukaemia	0.97 (0.90; 1.05)	0.94 (0.84; 1.06)	0.99 (0.89; 1.09)	1.09 (0.85; 1.40)	1.02 (0.90; 1.17)	0.93 (0.80; 1.07)	0.92 (0.80; 1.06)
Acute myeloid leukaemia	1.11 (0.98; 1.25)	1.03 (0.87; 1.22)	1.18 (1.01; 1.39)	1.29 (0.98; 1.70)	1.14 (0.90; 1.43)	1.12 (0.85; 1.48)	1.00 (0.82; 1.21)
Lymphoma	1.02 (0.88; 1.17)	1.00 (0.79; 1.26)	1.03 (0.86; 1.23)	1.77 (0.69; 4.58)	1.06 (0.76; 1.49)	1.04 (0.80; 1.35)	0.95 (0.78; 1.17)
Hodgkin Lymphoma	1.02 (0.69; 1.50)	0.80 (0.48; 1.34)	1.41 (0.78; 2.52)	n.a.	3.84 (0.52; 28.46)	1.13 (0.53; 2.41)	0.88 (0.54; 1.41)
Non-Hodgkin Lymphoma	1.07 (0.90; 1.29)	1.06 (0.80; 1.41)	1.08 (0.86; 1.34)	1.81 (0.40; 8.18)	1.02 (0.69; 1.51)	1.08 (0.78; 1.50)	1.08 (0.83; 1.40)
CNS tumours	0.93 (0.88; 0.98)	0.90 (0.83; 0.98)	0.94 (0.88; 1.01)	0.94 (0.80; 1.10)	1.01 (0.92; 1.11)	0.86 (0.78; 0.94)	0.91 (0.82; 1.01)
Malignant	0.95 (0.90; 1.00)	0.94 (0.86; 1.02)	0.96 (0.89; 1.03)	0.91 (0.77; 1.07)	1.04 (0.94; 1.15)	0.91 (0.83; 1.00)	0.92 (0.83; 1.02)
Non-malignant	0.84 (0.68; 1.04)	0.75 (0.55; 1.02)	0.93 (0.69; 1.24)	0.74 (0.42; 1.29)	0.69 (0.47; 1.01)	0.75 (0.50; 1.13)	1.24 (0.80; 1.90)
Neuroblastoma	1.05 (0.95; 1.15)	0.98 (0.84; 1.14)	1.09 (0.96; 1.24)	1.15 (0.88; 1.49)	1.04 (0.93; 1.17)	0.95 (0.73; 1.24)	0.94 (0.60; 1.47)
Retinoblastoma	0.96 (0.53; 1.75)	0.96 (0.42; 2.20)	1.00 (0.45; 2.21)	0.85 (0.34; 2.15)	1.10 (0.50; 2.41)	n.a.	n.a.
Renal tumours	1.07 (0.89; 1.30)	0.90 (0.69; 1.17)	1.30 (0.99; 1.71)	1.23 (0.83; 1.83)	1.03 (0.79; 1.36)	0.90 (0.62; 1.33)	1.46 (0.73; 2.92)
Hepatic tumours	0.96 (0.75; 1.23)	1.03 (0.68; 1.55)	0.93 (0.68; 1.26)	0.92 (0.46; 1.84)	0.99 (0.69; 1.42)	0.78 (0.44; 1.41)	1.11 (0.62; 1.99)
Bone tumours	0.94 (0.84; 1.05)	0.97 (0.83; 1.13)	0.90 (0.77; 1.06)	2.54 (0.27; 22.18)	0.68 (0.46; 1.01)	0.85 (0.69; 1.06)	0.99 (0.87; 1.14)
Soft tissue sarcomas	1.00 (0.91; 1.10)	0.98 (0.85; 1.13)	1.01 (0.89; 1.15)	0.97 (0.74; 1.28)	0.81 (0.68; 0.98)	0.99 (0.80; 1.24)	1.15 (0.98; 1.33)

Germ cell tumours	1.33 (0.99; 1.78)	0.97 (0.64; 1.47)	1.79 (1.18; 2.74)	1.38 (0.64; 2.95)	1.56 (0.71; 3.42)	2.40 (0.71; 8.06)	1.18 (0.82; 1.70)
Epithelial tumours and melanomas	1.09 (0.84; 1.42)	1.23 (0.82; 1.84)	1.01 (0.71; 1.44)	0.40 (0.02; 7.16)	1.01 (0.56; 1.83)	1.13 (0.60; 2.11)	1.13 (0.81; 1.59)
Other unspecified neoplasms	1.32 (0.57; 3.04)	0.56 (0.15; 2.05)	2.66 (0.46; 15.29)	n.a.	0.42 (0.09; 1.87)	n.a.	n.a.
Diagnostic period[d]							
1997-2001	1.01 (0.96; 1.06)	1.03 (0.96; 1.12)	0.99 (0.92; 1.06)	1.11 (0.94; 1.31)	1.00 (0.91; 1.10)	0.96 (0.86; 1.06)	1.02 (0.93; 1.11)
2002-2006	1.00 (0.94; 1.06)	0.90 (0.82; 0.98)	1.08 (1.00; 1.17)	1.08 (0.90; 1.29)	1.03 (0.92; 1.14)	0.95 (0.84; 1.06)	0.97 (0.87; 1.08)
2007-2011	1.03 (0.96; 1.10)	1.01 (0.91; 1.12)	1.05 (0.96; 1.14)	1.16 (0.95; 1.41)	1.04 (0.92; 1.18)	0.94 (0.82; 1.07)	1.07 (0.85; 1.20)
2012-2016	0.96 (0.89; 1.02)	0.88 (0.80; 0.98)	1.02 (0.92; 1.11)	1.01 (0.82; 1.23)	0.98 (0.87; 1.11)	0.92 (0.81; 1.05)	0.95 (0.83; 1.07)
Birth cohort[e]							
1982-1989	1.01 (0.93; 1.09)	1.12 (0.99; 1.27)	0.92 (0.82; 1.02)			1.04 (0.82; 1.32)	1.00 (0.92; 1.09)
1990-1999	1.01 (0.96; 1.05)	0.93 (0.87; 1.00)	1.07 (1.00; 1.14)	1.13 (0.92; 1.39)	1.03 (0.94; 1.12)	0.95 (0.88; 1.03)	1.02 (0.94; 1.11)
2000-2009	0.99 (0.94; 1.04)	0.94 (0.87; 1.02)	1.02 (0.95; 1.09)	1.10 (0.97; 1.25)	1.02 (0.94; 1.11)	0.91 (0.83; 1.01)	0.96 (0.84; 1.11)
2010-2016	1.00 (0.90; 1.11)	0.91 (0.78; 1.06)	1.07 (0.93; 1.23)	1.05 (0.88; 1.25)	0.96 (0.84; 1.09)	1.16 (0.74; 1.83)	
Place of residence[f,g]							
Urban	0.99 (0.96; 1.03)	0.95 (0.90; 1.00)	1.03 (0.98; 1.08)	1.09 (0.98; 1.21)	1.00 (0.93; 1.06)	0.94 (0.88; 1.01)	0.99 (0.93; 1.06)
Rural	1.02 (0.96; 1.08)	1.00 (0.91; 1.09)	1.03 (0.96; 1.12)	1.08 (0.89; 1.31)	1.04 (0.93; 1.16)	0.96 (0.85; 1.07)	1.04 (0.93; 1.15)

[a]Hazard ratios > 1 indicate that a higher mortality rate was associated with higher deprivation.
[b]HRs are expressed per 0.2 units (in accordance to the interquartile range/ standard deviation of the continuous variable GISD score)
[c]HRs were adjusted for year of diagnosis, year of birth and place of residence
[d]HRs were adjusted for year of birth and place of residence
[e]HRs were adjusted for year of diagnosis and place of residence
[f]place of residence was classified as either urban or rural area
[g]HRs were adjusted for year of diagnosis and year of birth
Abbreviations: CI, confidence interval; CNS, central nervous system; n.a., not applicable (due to small numbers)

Table S4: Adjusted hazard ratios with 95% confidence intervals (CI) of the association between absolute area-based socioeconomic deprivation (AASD) and 10-year overall survival from childhood cancer in Germany: additional analysis (Western German federal states only)

	Hazard ratio (95% CI)[a,b]						
	Total	Female	Male	<1 year	1-4 years	5-9 years	10-14 years
Diagnostic group/ cancer type							
All cancer types	1.01 (0.97; 1.05)	0.96 (0.91; 1.02)	1.04 (0.99; 1.10)	1.07 (0.96; 1.20)	1.01 (0.95; 1.08)	0.96 (0.89; 1.03)	1.02 (0.95; 1.09)
Leukaemia	1.03 (0.96; 1.11)	1.00 (0.90; 1.12)	1.05 (0.95; 1.16)	1.07 (0.89; 1.30)	1.14 (0.99; 1.30)	0.99 (0.86; 1.15)	0.96 (0.84; 1.10)
Lymphoblastic leukaemia	0.99 (0.90; 1.08)	0.99 (0.86; 1.14)	0.98 (0.87; 1.11)	1.02 (0.76; 1.35)	1.14 (0.97; 1.35)	0.93 (0.78; 1.10)	0.89 (0.75; 1.06)
Acute myeloid leukaemia	1.04 (0.90; 1.20)	0.91 (0.74; 1.13)	1.17 (0.96; 1.44)	1.20 (0.86; 1.69)	1.10 (0.82; 1.49)	1.05 (0.75; 1.48)	0.89 (0.70; 1.14)
Lymphoma	0.99 (0.83; 1.18)	0.91 (0.68; 1.22)	1.05 (0.83; 1.31)	3.02 (0.86; 10.57)	0.92 (0.60; 1.41)	0.96 (0.70; 1.33)	0.97 (0.75; 1.26)
Hodgkin Lymphoma	1.06 (0.65; 1.74)	0.87 (0.48; 1.60)	1.46 (0.66; 3.22)	n.a.	n.a.	0.82 (0.31; 2.23)	1.03 (0.57; 1.84)
Non-Hodgkin Lymphoma	1.05 (0.84; 1.32)	0.93 (0.65; 1.34)	1.14 (0.85; 1.53)	6.16 (0.51; 75.02)	0.84 (0.52; 1.34)	1.07 (0.70; 1.61)	1.12 (0.79; 1.57)
CNS tumours	0.96 (0.90; 1.02)	0.92 (0.83; 1.02)	0.98 (0.90; 1.07)	0.94 (0.77; 1.15)	1.03 (0.92; 1.16)	0.89 (0.80; 1.00)	0.98 (0.86; 1.12)
Malignant	0.97 (0.91; 1.04)	0.95 (0.86; 1.06)	0.98 (0.90; 1.07)	0.91 (0.74; 1.12)	1.06 (0.94; 1.20)	0.92 (0.82; 1.03)	0.98 (0.86; 1.11)
Non-malignant	0.86 (0.66; 1.12)	0.67 (0.46; 0.98)	1.04 (0.72; 1.51)	0.94 (0.48; 1.82)	0.63 (0.40; 0.99)	0.73 (0.44; 1.22)	1.36 (0.76; 2.43)
Neuroblastoma	0.98 (0.87; 1.10)	0.94 (0.78; 1.13)	0.98 (0.85; 1.14)	1.06 (0.76; 1.46)	0.98 (0.86; 1.12)	0.84 (0.60; 1.16)	0.94 (0.50; 1.75)
Retinoblastoma	1.03 (0.52; 2.01)	1.29 (0.52; 3.25)	0.90 (0.34; 2.35)	1.19 (0.42; 3.42)	0.95 (0.38; 2.39)	n.a.	n.a.
Renal tumours	1.08 (0.86; 1.36)	0.90 (0.65; 1.22)	1.32 (0.94; 1.86)	1.30 (0.80; 2.08)	0.99 (0.71; 1.39)	0.94 (0.61; 1.45)	1.84 (0.47; 7.27)
Hepatic tumours	1.22 (0.91; 1.62)	1.28 (0.79; 2.08)	1.17 (0.82; 1.68)	1.34 (0.60; 3.01)	1.32 (0.85; 2.04)	0.72 (0.39; 1.31)	1.83 (0.83; 4.00)
Bone tumours	1.00 (0.87; 1.14)	0.98 (0.81; 1.19)	1.01 (0.84; 1.21)	n.a.	0.71 (0.47; 1.06)	1.01 (0.77; 1.32)	1.04 (0.88; 1.23)
Soft tissue sarcomas	1.02 (0.90; 1.14)	1.02 (0.85; 1.22)	1.01 (0.86; 1.18)	0.91 (0.66; 1.25)	0.86 (0.69; 1.07)	1.04 (0.80; 1.34)	1.21 (0.99; 1.47)

Germ cell tumours	1.22 (0.85; 1.74)	0.86 (0.53; 1.42)	1.84 (1.07; 3.11)	1.49 (0.61; 3.60)	1.05 (0.38; 2.92)	2.65 (0.58; 12.15)	1.11 (0.71; 1.72)
Epithelial tumours and melanomas	1.21 (0.87; 1.68)	1.60 (0.95; 2.70)	1.03 (0.66; 1.60)	0.48 (0.03; 7.91)	0.78 (0.39; 1.57)	1.56 (0.68; 3.58)	1.36 (0.89; 2.09)
Other unspecified neoplasms	0.83 (0.29; 2.35)	0.45 (0.12; 1.67)	3.74 (0.34; 41.14)	n.a.	0.57 (0.15; 2.14)	n.a.	n.a.
Diagnostic period[c]							
1997-2001	0.99 (0.92; 1.07)	0.98 (0.88; 1.10)	0.99 (0.90; 1.10)	1.12 (0.89; 1.41)	0.95 (0.83; 1.08)	0.97 (0.84; 1.11)	1.03 (0.89; 1.19)
2002-2006	1.01 (0.94; 1.09)	0.91 (0.82; 1.02)	1.10 (0.99; 1.21)	1.06 (0.86; 1.32)	1.07 (0.94; 1.22)	0.87 (0.85; 1.14)	0.95 (0.83; 1.09)
2007-2011	1.07 (0.99; 1.15)	1.04 (0.93; 1.17)	1.09 (0.99; 1.20)	1.10 (0.86; 1.39)	1.08 (0.94; 1.24)	1.00 (0.87; 1.16)	1.16 (1.02; 1.32)
2012-2016	0.96 (0.89; 1.03)	0.91 (0.81; 1.02)	1.00 (0.90; 1.11)	1.02 (0.81; 1.28)	1.00 (0.88; 1.14)	0.89 (0.77; 1.04)	0.96 (0.83; 1.11)
Birth cohort[d]							
1982-1989	1.00 (0.88; 1.13)	1.07 (0.88; 1.31)	0.93 (0.79; 1.11)			0.88 (0.60; 1.29)	1.02 (0.89; 1.16)
1990-1999	1.01 (0.96; 1.07)	0.92 (0.85; 1.01)	1.09 (1.01; 1.18)	1.02 (0.76; 1.36)	0.97 (0.86; 1.09)	1.00 (0.90; 1.11)	1.04 (0.94; 1.14)
2000-2009	1.01 (0.95; 1.07)	0.99 (0.90; 1.08)	1.02 (0.94; 1.11)	1.10 (0.95; 1.29)	1.06 (0.96; 1.17)	0.94 (0.84; 1.05)	0.98 (0.85; 1.14)
2010-2016	1.01 (0.90; 1.13)	0.93 (0.79; 1.10)	1.08 (0.93; 1.26)	1.05 (0.86; 1.18)	0.99 (0.86; 1.13)	1.13 (0.67; 1.92)	
Place of residence[e,f]							
Urban	1.01 (0.96; 1.05)	0.96 (0.90; 1.02)	1.05 (0.99; 1.11)	1.08 (0.95; 1.22)	1.00 (0.93; 1.08)	0.95 (0.88; 1.04)	1.03 (0.95; 1.12)
Rural	1.01 (0.94; 1.08)	0.97 (0.87; 1.09)	1.03 (0.94; 1.13)	1.05 (0.83; 1.33)	1.04 (0.92; 1.18)	0.98 (0.85; 1.12)	0.99 (0.87; 1.13)

[a]Hazard Ratios are expressed per 0.3 units (in accordance to the standard deviation/ interquartile range of the continuous variable AASD score)
[b]adjusted for year of birth, year of diagnosis, place of residence
[c]adjusted for year of birth and place of residence
[d]adjusted for year of diagnosis and place of residence
[e]place of residence was classified as either urban or rural
[f]adjusted for year of birth, year of diagnosis
Abbreviations: CI, confidence interval; CNS, central nervous system; AASD, absolute area based socioeconomic deprivation

Table S5: Adjusted hazard ratios with 95% confidence intervals (CI) of the association between single SES indicators and 10-year overall survival from childhood cancer in Germany: additional analysis (Western German federal states only)

	Hazard ratio (95% CI)[a]		
	Household net income[b]	Unemployment[c]	1 / Employees with a university degree[d]
Diagnostic group/ cancer type			
All cancer types	1.01 (0.98; 1.05)	1.03 (0.98; 1.09)	1.00 (0.98; 1.03)
Leukaemia	1.00 (0.93; 1.07)	1.11 (1.00; 1.24)	1.01 (0.96; 1.06)
Lymphoblastic leukaemia	1.05 (0.97; 1.14)	1.15 (1.01; 1.32)	0.96 (0.90; 1.02)
Acute myeloid leukaemia	0.97 (0.85; 1.11)	0.95 (0.76; 1.18)	1.10 (1.00; 1.21)
Lymphoma	1.10 (0.94; 1.29)	1.02 (0.79; 1.31)	0.91 (0.80; 1.03)
Hodgkin Lymphoma	1.18 (0.77; 1.82)	1.32 (0.69; 2.55)	0.95 (0.69; 1.32)
Non-Hodgkin Lymphoma	1.03 (0.84; 1.26)	1.13 (0.83; 1.56)	0.94 (0.80; 1.09)
CNS tumours	1.04 (0.98; 1.11)	0.98 (0.89; 1.08)	0.99 (0.95; 1.03)
Malignant	1.06 (1.00; 1.13)	0.99 (0.90; 1.09)	1.00 (0.95; 1.04)
Non-malignant	1.09 (0.87; 1.38)	0.98 (0.67; 1.42)	0.93 (0.79; 1.11)
Non-CNS solid tumours	0.99 (0.94; 1.05)	1.04 (0.94; 1.14)	1.02 (0.97; 1.06)

[a]adjusted for year of birth, year of diagnosis, place of residence

[b]Hazard Ratios are expressed per EUR 250

[c]Hazard Ratios are expressed per 40 (persons per 1000 inhabitants)

[d]Hazard Ratios are expressed per 0.05%

Abbreviations: CI, confidence interval; CNS, central nervous system

Removing the normalisation step of the original GISD

To enable longitudinal comparisons, we recalculated the GISD by using the original statistical codes and input data as published by the GISD authors. The published version of the GISD is calculated by first building domain-specific scores for income, occupation, and education. Separately for each calendar year, these domain-specific scores are summed, and the sum is then normalised to the interval [0, 1]. This is accomplished by subtracting the minimum sum score among all municipalities from each municipality's sum score, and then dividing the resulting value by the range of the sum score over all municipalities. As such, the GISD score indicates the socioeconomic deprivation of a given municipality relative to all others in the same year. However, the same GISD value from different years may refer to different absolute levels of socioeconomic deprivation when all municipalities change in deprivation without affecting their rank order. Therefore, the AASD was calculated exactly as the GISD except that we eliminated the calendar-year specific normalisation step for the sum of domain-specific scores.

For the AASD, we removed the normalisation step, such that the AASD is just the sum of the domain-specific scores income, occupation, and education. Figure S7 compares the AASD and the GISD. For each year, AASD and GISD have near perfect correlation, but the absolute level of the AASD may differ from the GISD.

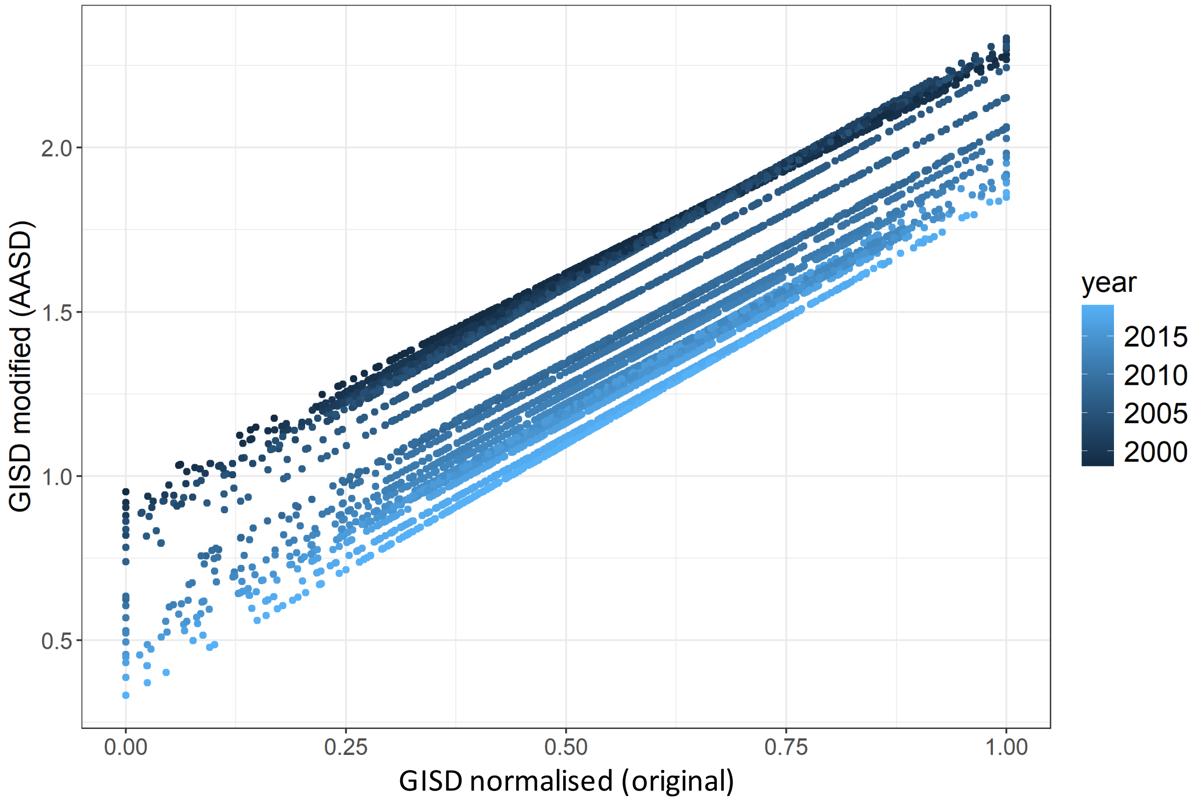

Figure S7: Scatterplot of the AASD against the GISD with the colour-wash indicating the calendar year. Each point represents one municipality in a separate year.
